# Soma-germline communication drives sex maintenance in the *Drosophila* testis

**DOI:** 10.1093/nsr/nwae215

**Published:** 2024-06-22

**Authors:** Rui Zhang, Peiyu Shi, Shuyang Xu, Zhe Ming, Zicong Liu, Yuanyuan He, Junbiao Dai, Erika Matunis, Jin Xu, Qing Ma

**Affiliations:** Shenzhen Key Laboratory of Synthetic Genomics, Guangdong Provincial Key Laboratory of Synthetic Genomics, Key Laboratory of Quantitative Synthetic Biology, Shenzhen Institute of Synthetic Biology, Shenzhen Institute of Advanced Technology, Chinese Academy of Sciences, Shenzhen 518055, China; State Key Laboratory of Biocontrol, School of Life Sciences, Sun Yat-Sen University, Guangzhou 510275, China; Shenzhen Key Laboratory of Synthetic Genomics, Guangdong Provincial Key Laboratory of Synthetic Genomics, Key Laboratory of Quantitative Synthetic Biology, Shenzhen Institute of Synthetic Biology, Shenzhen Institute of Advanced Technology, Chinese Academy of Sciences, Shenzhen 518055, China; Shenzhen Key Laboratory of Synthetic Genomics, Guangdong Provincial Key Laboratory of Synthetic Genomics, Key Laboratory of Quantitative Synthetic Biology, Shenzhen Institute of Synthetic Biology, Shenzhen Institute of Advanced Technology, Chinese Academy of Sciences, Shenzhen 518055, China; Shenzhen Key Laboratory of Synthetic Genomics, Guangdong Provincial Key Laboratory of Synthetic Genomics, Key Laboratory of Quantitative Synthetic Biology, Shenzhen Institute of Synthetic Biology, Shenzhen Institute of Advanced Technology, Chinese Academy of Sciences, Shenzhen 518055, China; Shenzhen Key Laboratory of Synthetic Genomics, Guangdong Provincial Key Laboratory of Synthetic Genomics, Key Laboratory of Quantitative Synthetic Biology, Shenzhen Institute of Synthetic Biology, Shenzhen Institute of Advanced Technology, Chinese Academy of Sciences, Shenzhen 518055, China; Shenzhen Key Laboratory of Synthetic Genomics, Guangdong Provincial Key Laboratory of Synthetic Genomics, Key Laboratory of Quantitative Synthetic Biology, Shenzhen Institute of Synthetic Biology, Shenzhen Institute of Advanced Technology, Chinese Academy of Sciences, Shenzhen 518055, China; Department of Cell Biology, Johns Hopkins University School of Medicine, Baltimore, MD 21205, USA; State Key Laboratory of Biocontrol, School of Life Sciences, Sun Yat-Sen University, Guangzhou 510275, China; Shenzhen Key Laboratory of Synthetic Genomics, Guangdong Provincial Key Laboratory of Synthetic Genomics, Key Laboratory of Quantitative Synthetic Biology, Shenzhen Institute of Synthetic Biology, Shenzhen Institute of Advanced Technology, Chinese Academy of Sciences, Shenzhen 518055, China

**Keywords:** *Drosophila* testis, somatic stem cell sex conversion, single-cell sequencing, soma-germline communication, insulin signaling

## Abstract

In adult gonads, disruption of somatic sexual identity leads to defective gametogenesis and infertility. However, the underlying mechanisms by which somatic signals regulate germline cells to achieve proper gametogenesis remain unclear. In our previous study, we introduced the *chinmo^Sex Transformation^* (*chinmo^ST^*) mutant *Drosophila* testis phenotype as a valuable model for investigating the mechanisms underlying sex maintenance. In *chinmo^ST^* testes, depletion of the Janus Kinase-Signal Transducer and Activator of Transcription downstream effector Chinmo from somatic cyst stem cells (CySCs) feminizes somatic cyst cells and arrests germline differentiation. Here, we use single-cell RNA sequencing to uncover *chinmo^ST^*-specific cell populations and their transcriptomic changes during sex transformation. Comparative analysis of intercellular communication networks between wild-type and *chinmo^ST^* testes revealed disruptions in several soma-germline signaling pathways in *chinmo^ST^* testes. Notably, the insulin signaling pathway exhibited significant enhancement in germline stem cells (GSCs). Chinmo cleavage under targets and tagmentation (CUT&Tag) assay revealed that Chinmo directly regulates two male sex determination factors, *doublesex* (*dsx*) and *fruitless* (*fru*), as well as *Ecdysone-inducible gene L2* (*ImpL2*), a negative regulator of the insulin signaling pathway. Further genetic manipulations confirmed that the impaired gametogenesis observed in *chinmo^ST^* testes was partly contributed by dysregulation of the insulin signaling pathway. In summary, our study demonstrates that somatic sex maintenance promotes normal spermatogenesis through Chinmo-mediated conserved sex determination and the insulin signaling pathway. Our work offers new insights into the complex mechanisms of somatic stem cell sex maintenance and soma-germline communication at the single-cell level. Additionally, our discoveries highlight the potential significance of stem cell sex instability as a novel mechanism contributing to testicular tumorigenesis.

## Teaser

Single-cell RNA-seq reveals how Chinmo and soma-germline communication ensure male stem cell sex maintenance in the *Drosophila* testis.

## INTRODUCTION

The production of distinct male and female gametes is crucial for sexual reproduction. Although significant emphasis is placed on germ cells, somatic gonadal cells also play an indispensable role in ensuring proper gametogenesis [1,2]. In *Drosophila* gonads, somatic-germline sex mismatch experiments have revealed that somatic sexual identity is pivotal for normal differentiation and sex determination of germline cells during embryonic development [1–3]. Germline sex determination is influenced by signaling from the surrounding somatic cells. One critical signaling pathway involved in this non-autonomous regulation is the Janus Kinase-Signal Transducer and Activator of Transcription (JAK-STAT) pathway [4]. In many organisms, cellular sex identity, established during embryonic development, was thought to be unalterable; however, recent work challenges this notion by revealing that somatic sexual identity can be transdifferentiated in adult gonads [5–11]. This suggests that somatic sexual identity is actively maintained during adulthood or the postnatal period. Disruption of this maintenance can also lead to defective gametogenesis and infertility. For instance, in adult mice, sexual transformation of the Sertoli cells through conditional loss of Doublesex (Dsx)/Mab-3-Related Transcription Factor 1 (DMRT1) results in feminized germ cells [6]. Similarly, in adult *Drosophila* gonads, sexual transformation of somatic stem cells leads to the formation of tumorous mitotic germline [9–11]. Nevertheless, despite the significant advances in understanding sex determination mechanisms and the influence of somatic sexual identity on germ cells during embryonic development, the molecular mechanisms underlying the maintenance of sexual identity in adulthood and the associated signals between somatic cells and germ cells remain largely unexplored.

We initially reported a mutant *Drosophila* testis phenotype where CySCs undergo sex conversion in adulthood, offering an excellent model to investigate the soma-germline communication involved in somatic sex maintenance [11]. Previous genetic complementation and rescue experiments have elucidated the pivotal role of chronologically inappropriate morphogenesis (Chinmo), a key effector of the JAK-STAT signaling pathway, in the manifestation of this mutant phenotype [11]. A presumed regulatory mutation in *chinmo* specifically eliminates the expression of Chinmo only in CySCs and their progeny, resulting in their feminization. Consequently, we denoted this loss-of-function allele as *chinmo^Sex Transformation^* or *chinmo^ST^*. The precise genetic lesion in *chinmo^ST^* is unknown. Importantly, RNAi knockdown of *chinmo* in the CySC lineage phenocopies *chinmo^ST^* albeit with lower penetrance and a later phenotypic onset [11]. Therefore, we use *chinmo^ST^* in the current study. Previously, we morphologically characterized the feminized somatic cyst cells and validated our findings using the few available sex-specific somatic cell markers such as Dsx, the fly homologue of the crucial human male determination factor DMRT1. However, the lineage trajectory of these progressively transdifferentiating CySCs and genome-wide transcriptomic profiles of different somatic and germ cells remain unknown. Furthermore, whether and how the Chinmo-induced sexually transformed CySCs communicate with the germline is poorly understood.

In this study, we use a time series of single-cell RNA-sequencing (scRNA-seq) to characterize the transcriptional profiles for wild-type and *chinmo^ST^* testes. We identified *chinmo^ST^*-specific cell populations and transcriptomic changes corresponding to the phenotype. By comparing intercellular communication networks between wild-type and *chinmo^ST^* testes, we discovered disruptions in several soma-germline signaling pathways, including the insulin signaling pathway, in *chinmo^ST^* testes. Using the Chinmo CUT&Tag (cleavage under targets and tagmentation) assay, we found that some sex determination factors and insulin pathway components are directly regulated by Chinmo. Altogether, our study demonstrates that somatic sex maintenance promotes normal spermatogenesis, which is mediated by both conserved sex determination factors and the insulin signaling pathway.

## RESULTS

### Single-cell-resolution gene expression and cellular heterogeneity in the testis

**Figure 1.**
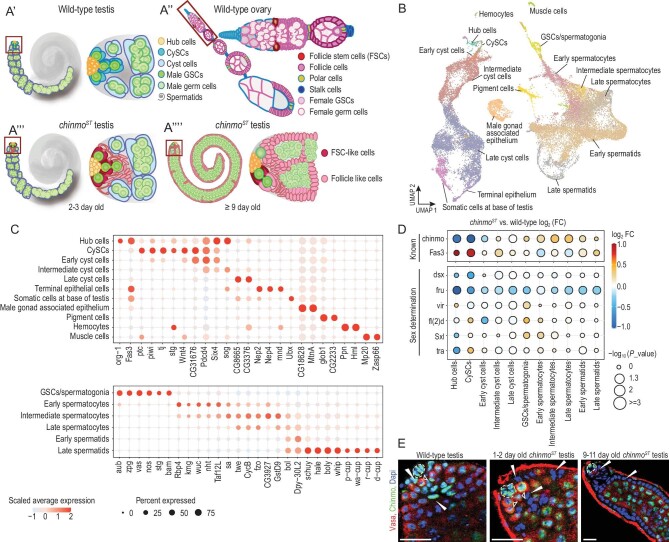
Identification and annotation of testis cell types by single-cell RNA-seq. (A′–A′′′′) Illustrations of a wild-type testis (A′), wild-type ovary (A′′), 2–3-day-old *chinmo^ST^* testis (A′′′) and an older *chinmo^ST^* testis (A′′′′). Apex of testis or ovary, red box. Within the wild-type testis apex, 8–15 hub cells and the surrounding stem cells constitute the male stem cell niche, producing signals that support both germline stem cells (GSCs) and somatic cyst stem cells (CySCs) for self-renewal and differentiation [45] (A′). GSCs undergo oriented mitotic divisions to produce the gonialblast (Gb) [46], 2-, 4-, 8- and 16-cell spermatogonial cysts. Each 16-cell cyst undergoes meiosis to generate mature spermatids [47]. GSCs are supported by CySCs. Each Gb is ensheathed by two differentiating squamous cyst cells, which both exit the cell cycle and continue increasing in size to support germ cells throughout spermatogenesis [48,49]. In the young *chinmo^ST^* testis (A′′′), CySCs and their early progeny lose male fate and adopt a follicle stem cell/progenitor-like cell identity. As flies age, Fas3-positive follicle stem cell/progenitor-like cells produce follicle-like cells, gradually displacing normal cyst cells from the niche. Germ cells, restricted to the testis lumen, overproliferate and arrest as spermatogonia, resulting in overproliferation of mitotic germ cells (DAPI-bright and BrdU/EdU-positive, with 1B1-positive spherical or short branching fusomes) [9,11,24,50] when the germline and somatic sex are mismatched. (B) Uniform manifold approximation and projection (UMAP) plot of the integrated scRNA-seq data set from the wild-type and *chinmo^ST^* adult testis scRNA-seq data sets with cell type annotations. (C) Dot plot showing scaled average expression of marker genes for somatic (upper plot) and germline (lower plot) cell types. (D) Dot plot showing *chinmo^ST^* versus wild-type cell-type-specific differential expression patterns for known gene markers and sex determination factors. (E) Chinmo staining at the apex of the testes of wild-type testis, and young and old *chinmo^ST^* testis. Vasa marks germline cells. DAPI marks nuclei. Dotted lines mark hub cells. Arrowheads mark somatic cyst cells. Hollow arrowheads mark GSCs. Scale bars = 20 μm. WT, wild-type.

In *chinmo^ST^* mutants, adult testes initially appear normal but progressively undergo somatic lineage feminization with age: squamous cyst cells transform into columnar epithelial cells resembling ovarian somatic follicle cells [11]. In addition, the germ cells in 7- to 9-day-old *chinmo^ST^* testes appear to arrest as early male germ cells (spermatogonia), resulting in overproliferation of mitotic germ cells [8,9,11] (Fig. 1A′–A′′′′, Fig. S1A). To investigate the sequential molecular changes across different cell types during the progression of the sex conversion phenotype in *chinmo^ST^* testes, we performed scRNA-seq on wild-type and *chinmo^ST^* testes across three time points (3–5, 6–8 and 9–11 days of adulthood) which yielded 26 549 high-quality cells (Fig. 1A′–A′′′′, Fig. S1C). In combination with unsupervised clustering analysis and previous knowledge of cell-type-specific marker genes [12–14], we identified 17 cell populations, including 11 somatic cell types and 6 germline cell types (Fig. 1B and C, Figs S1D and S2A–J, Supplementary Table S1). Furthermore, the alignment of our annotated cell types with those from the Fly Cell Atlas testis study [13] substantiates the consistency of cell-type classification across independent studies (Fig. S1E). As expected, the number of differentially expressed genes (DEGs) increases over time in *chinmo^ST^* testes compared to wild-type testes (Fig. S3A). This pattern aligns with the observed distal expansion of mutant somatic and germ cells with age [11] (Fig. S1C).

To compare the cell-type-specific gene expression patterns of *chinmo^ST^* and wild-type testes, we first analyzed the few known genes with predictable changes. We found that *chinmo* expression decreases dramatically in the CySC lineage and slightly increases over time in early germ cells in *chinmo^ST^* testes, as expected (Fig. 1D, Fig. S3B and D). This result is corroborated by Chinmo protein levels as assayed by immunostaining (Fig. 1E) [8,9,11]. Similarly, the somatic membrane adhesion marker *Fasciclin 3* (*Fas3*) is sequentially upregulated in the CySC lineage over time as expected [8,9,11] (Fig. 1D, Fig. S3C). We next analyzed DEGs involved in the canonical sex determination pathway. As expected, expression of the male-specific sex determination factors *doublesex* (*dsx*) and *fruitless* (*fru*) is downregulated [9,11], while expression of the female-specific sex determination regulator *female lethal d* (*fl(2)d*) [9] and *transformer* (*tra*) [3,9,15] is upregulated, in *chinmo^ST^* CySCs (Fig. 1D).

Taken together, our single-cell data are consistent with previous observations that Chinmo partially functions through the canonical sex determination pathway to promote the male identity of CySCs [9,11]. These findings serve as a foundation for investigating the molecular mechanisms underlying somatic CySCs feminization and germ cell tumorigenesis.

### Single-cell RNA-seq reveals transcriptomic changes in mutant-specific somatic cell populations

**Figure 2.**
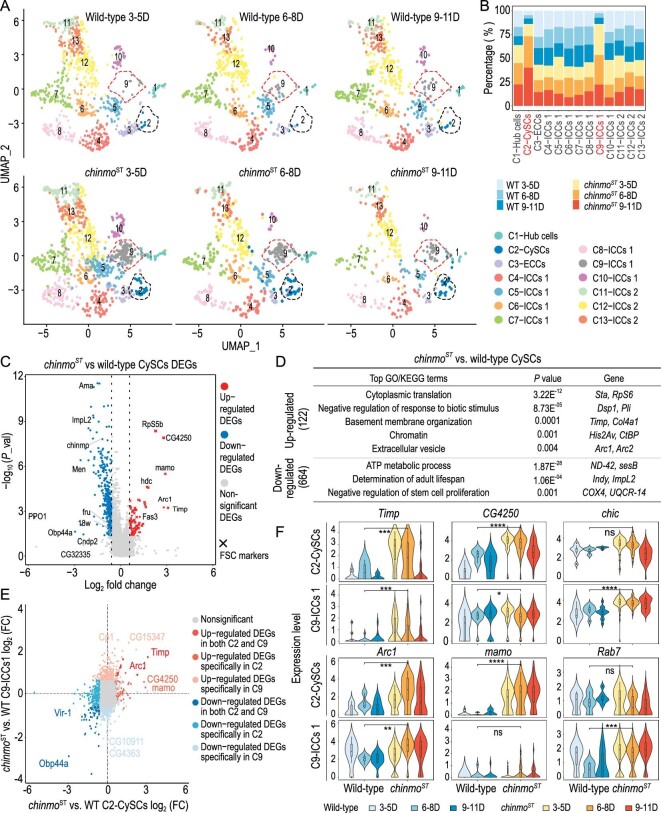
scRNA-seq of wild-type and *chinmo^ST^* mutant testes reveals mutant-specific somatic transcriptomic changes. (A) Time series UMAP plots of hub cells, CySCs, and early and intermediate cyst cells for wild-type and *chinmo^ST^* testes, which consist of 13 clusters. Dotted lines mark CySCs and C9-ICCs1, respectively. (B) Bar plot showing the percentage of *chinmo^ST^* and wild-type cells in each of the 13 somatic clusters over time. (C) Volcano plot of DEGs between *chinmo^ST^* and wild-type CySCs. Genes with Wilcoxon rank sum test *P* value < 0.05 and absolute value of fold change ≥ 1.5 are considered as DEGs, while others are considered non-significant DEGs. (D) Table showing top GO or KEGG terms (one-sided Fisher's exact test with Benjamini-Hochberg correction, adjusted *P* value < 0.05) enriched for up- and downregulated DEGs in *chinmo^ST^* versus wild-type CySCs (Wilcoxon rank sum test *P* value < 0.05, absolute value of fold change ≥ 1.5), respectively. (E) Scatter plot showing *chinmo^ST^* versus wild-type log_2_ fold change of gene expression in C2-CySCs (x-axis) and C9-ICCs1 (y-axis). (F) Violin plots showing expression of *Timp, Arc1, CG4250, mamo, chic* and *Rab7* in C2-CySCs and C9-ICCs1 over time. Differential expression analysis is performed by Wilcoxon rank sum test (*, *P* < 0.05; **, *P* < 0.01; ****, *P* < 0.0001; ns, non-significant).

We next focused on transcriptomic changes in the somatic cyst lineage over time, which undergoes progressive feminization in *chinmo^ST^* testes. To get a better resolution of cellular differences, we selected hub cells, CySCs, early cyst cells (ECCs) and intermediate cyst cells (ICCs) for further subclustering analysis (Fig. S4A–D, see ‘Methods’), which yielded 13 clusters (Fig. 2A, Fig. S4C and D).

Among these 13 clusters, we found that cluster 2 and cluster 9 were largely contributed by *chinmo^ST^* testes (Fig. 2A and B). Furthermore, these two clusters exhibited more overall similarities to wild-type ovarian somatic cells [16] than to wild-type testes (Fig. S4E and F). Notably, the pseudobulk transcriptomes of cluster 2 had a higher global similarity to wild-type ovarian follicle stem cells (FSCs) (with a correlation coefficient of 0.97) than to wild-type CySCs (with a correlation coefficient of 0.93) (Fig. S4F). Therefore, we concluded that clusters 2 and 9 represent feminized CySCs and a mutant-specific cyst cell population, respectively, which we refer to as C2-CySCs and C9-ICCs1. Comparison of DEGs between *chinmo^ST^* C2-CySCs and wild-type CySCs to genes expressed in wild-type FSCs revealed that most DEGs (74.5%) in *chinmo^ST^* CySCs trended toward female expression patterns (Fig. S5). Only 25.5% of DEGs are differentially expressed compared to wild-type female cells (Fig. S5). These results indicate that *chinmo^ST^* C2-CySCs and cluster-9 cyst cells indeed exhibit clear signs of feminization (Figs S4E and F, S5).

We next examined the molecular features of the two feminized *chinmo^ST^*-specific clusters, C2-CySCs and C9-ICCs1, by differential expression analysis (Fig. 2C and D, Fig. S4G). As the *chinmo^ST^* phenotype progresses, these clusters exhibit sequential transcriptomic changes (Fig. 2F, Fig. S3A–C, E–G). With time, the number of up- and downregulated DEGs increases in C2-CySCs compared to their counterparts from wild-type testes (Fig. S3A). Similarly, the number of upregulated DEGs increases in C2-CySCs’ descendants, while the number of downregulated DEGs initially increases and then decreases with time (Fig. S3A). Specifically, C2-CySCs and the *chinmo^ST^*-specific C9-ICCs1 share similar transcriptomic changes including the downregulation of mitochondrial functions and the progressive upregulation of ovarian follicle cell markers such as *Tissue inhibitor of metalloproteases* (*Timp*) and *Activity-regulated cytoskeleton associated protein 1* (*Arc1*) [16] (Fig. 2C, D and F). Also, the *chinmo^ST^* C2-CySCs show significant upregulation of follicle stem cell markers such as *CG4250* [16] (Fig. 2C, D and F). Functional enrichment of DEGs indicated that *chinmo^ST^* C2-CySCs are highly primed for cellular proliferation and intercellular communications, potentially contributing to the formation of actively dividing follicle-like cells and disturbed germ cells (Fig. 2D). On the other hand, *chinmo^ST^* C9-ICCs1 shows specific upregulation in lysosome activity, vesicle-mediated transport and cytoskeletal activity, along with a specific downregulation in spermatid differentiation (Fig. S4G–I), which is consistent with the identity of these cells as a mitotically active epithelial monolayer that loses its capacity to support germ cell development. Examples of representative upregulated genes include the actin monomer binding protein *chickadee* (*chic*), the vesicle trafficking regulator *Rab7*, and the cell adhesion factor *Matrix metalloproteinase 1* (*Mmp1*) [16] (Fig. 2F, Fig. S4G). Conversely, the late cyst cell protein *α-Tubulin at 85E* (*αTub85E*), which marks late cyst cells supporting developing spermatid cysts [17], and the sperm DNA condensation factor *Male-specific transcript 77F* (*Mst77F*) [18] are both downregulated (Fig. S4G). These results suggest that *chinmo^ST^* C9-ICCs1 are sex-transformed cyst cells resembling differentiated follicle cells, and that their molecular changes could affect normal spermatid development. Furthermore, we conducted a thorough comparison of each annotated cyst cell population with the sorted bulk mutant cyst cells [8] and found substantial overlaps in DEGs across most populations (Fig. S6). However, we observed an exception in the CySC population, which might be under-represented in the sorted cells analyzed by Grmai *et al.* 2021.

In summary, *chinmo^ST^* testes feature a distinct increase in ovarian follicle-like cells that we confirmed and profiled by scRNA-seq. There are two major *chinmo^ST^*-specific somatic cyst cell subpopulations: (i) 1%–2% of *chinmo^ST^* CySCs that resemble follicle stem cells, (ii) the collection of feminized somatic cells that likely originate from the feminized CySCs and morphologically resemble epithelial follicle-like cells [8,9,11]. Multiple biological processes accompany the sex conversion of CySCs and the formation of their follicle-like progeny, such as stem cell proliferation, ribosome assembly, vesicle-mediated transport and metabolic changes.

### Germline stem cells/spermatogonia and early spermatocytes encysted by mutant soma show confused sexual identity

In *Drosophila* gonads, manipulated somatic sexual microenvironments can influence the sexual cell fate stability of germ cells [19–21]. In *chinmo^ST^* testes, germ cells overproliferate and arrest as early male germ cells (spermatogonia), as indicated by their morphology and expression of the male-specific early germ cell marker M5-4 [8,9,11]. Therefore, we next turned our attention to the germline lineage to investigate the molecular features of arrested germ cells while neighboring somatic cells become feminized.

**Figure 3.**
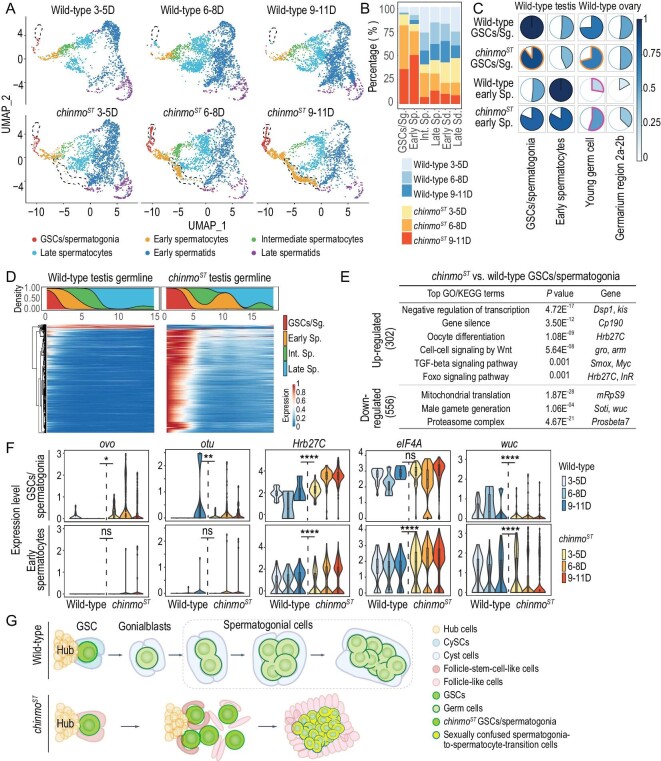
scRNA-seq reveals mutant-specific germline cell populations and molecular changes. (A) Time series UMAP plots of wild-type and *chinmo^ST^* germline cells. Cells are colored by cell types. Dotted lines mark *chinmo^ST^*-specific GSCs/spermatogonia and early spermatocytes, respectively. (B) Bar plot showing percentage of each sample in different germline cell types. (C) Pie charts showing Pearson correlation coefficients of pseudobulk transcriptomes between wild-type/*chinmo^ST^* testis GSCs/spermatogonia and early germ cells from wild-type testes/ovaries (top), and between wild-type/*chinmo^ST^* early spermatocytes and early germ cells from wild-type testes/ovaries (bottom). (D) Heatmap showing the smoothed expression patterns of DEGs (Wald test with Benjamini-Hochberg correction, adjusted *P* value < 0.05, absolute value of fold change > 1) between *chinmo^ST^* and wild-type testes along the trajectory from GSCs to late spermatocytes (bottom), together with the smoothed proportions of annotated cells along the trajectory (top). (E) Table showing top GO or KEGG terms (one-sided Fisher's exact test with Benjamini-Hochberg correction, adjusted *P* value < 0.05) enriched for up- and downregulated DEGs in *chinmo^ST^* versus wild-type GSCs/spermatogonia (Wilcoxon rank sum test *P* value < 0.05, absolute value of fold change ≥ 1.5), respectively. (F) Violin plots showing expression of *ovo, otu, Hrb27C, elF4A* and *wuc* in early testes germ cells over time. Differential expression analysis is performed by Wilcoxon rank sum test (*, *P* < 0.05; **, *P* < 0.01; ****, *P* < 0.0001; ns, non-significant). (G) Working model of how germ cells respond to adjacent sex-converted CySCs and their descendant follicle-like cells mediated by loss of Chinmo. WT, wild-type. Sg, spermatogonia. Sp, spermatocytes. Sd, spermatids. Int, intermediate.

Six major germ cell types were subset for further analysis, including germline stem cells (GSCs)/spermatogonia, early spermatocytes, intermediate spermatocytes, late spermatocytes, early spermatids and late spermatids (Fig. 3A, Fig. S7A and B). Two subpopulations are specific to *chinmo^ST^* testes, which were annotated as GSCs/spermatogonia and early spermatocytes, respectively (Fig. 3A). Correspondingly, the percentage of GSCs/spermatogonia and early spermatocytes increased substantially in *chinmo^ST^* testes (Fig. 3B). The number of cells in these two cell types also increased progressively with age in *chinmo^ST^* testes (Fig. 3B). In contrast, the proportion of late-stage germ cell types, such as early and late spermatids, decreased with time in *chinmo^ST^* testes. These findings are consistent with prior phenotypic observations that GSCs/spermatogonia in *chinmo^ST^* testes proliferate substantially and their descendant spermatogonia-to-spermatocyte-transition cells are arrested at an early stage and unable to differentiate into mature sperm cells [8,9,11].

To investigate the sexual identity of the germ cell lineage in *chinmo^ST^* testes, we compared transcriptomes for all the germ cells among *chinmo^ST^* testes, wild-type testes and ovaries [13], both at the aggregate and cell-type levels (Fig. S7C and D, see ‘Methods’). Globally, as the *chinmo^ST^* phenotype progresses, the RNA profiles in *chinmo^ST^* germ cells show sequential transcriptomic changes that resemble wild-type ovarian germ cells (Fig. S7C). Changes are not uniform across the germline lineage. For example, *chinmo^ST^* GSCs/spermatogonia are more similar to wild-type GSCs/spermatogonia than to ovarian germ cells (Fig. 3C, Fig. S7D). By contrast, *chinmo^ST^* early spermatocytes display increased similarity with those of the ovarian germline (Fig. 3C, Fig. S7D). This suggests that a potential sex transition point is likely from spermatogonia to early spermatocytes during the process of sex conversion. We also found heterogeneity within early spermatocytes, depicting a ‘mosaic’ pattern of sex conversion (Fig. S8A and B). We further performed trajectory analysis of wild-type and *chinmo^ST^* germ cells (see ‘Methods’) to track their dynamics. RNA velocity results revealed that *chinmo^ST^* GSCs/spermatogonia experience dramatic transcriptional changes, while *chinmo^ST^* early spermatocytes sustain small transcriptional changes and fail to differentiate into mature spermatocytes (Fig. S7E). Conventional trajectory inference revealed that along the inferred trajectory of germ cells, 3767 DEGs are dynamically expressed in *chinmo^ST^* testes, with the majority of these DEGs being strongly upregulated during the development process from GSCs/spermatogonia to early spermatocytes (Fig. 3D, Fig. S7F). This indicates that transcriptomic changes in *chinmo^ST^* germ cells are initiated and take effect at a very early stage, likely within the GSCs.

To further examine the molecular features of mutant GSCs/spermatogonia and early spermatocytes, we next performed differential expression analysis (Fig. 3E and F, Fig. S7G–J). In *chinmo^ST^* GSCs/spermatogonia, the expression patterns of genes involved in spermatogenesis like *PFTAIRE-interacting factor 1A* (*Pif1a*) [22], *aubergine* (*aub*) and *boule* (*bol*) [13,23] (Fig. S9A–C), are consistent with the previously reported male-specific M5-4 (Esg) expression in *chinmo^ST^* GSCs/spermatogonia [11]. In *chinmo^ST^* GSCs/spermatogonia, there are 302 upregulated genes enriched in multiple signaling pathways, such as Wnt, Foxo and Transforming Growth Factor-β (TGF-β) pathways (Fig. 3E). This indicated that soma-germline interactions might be disrupted due to the feminization of somatic cyst cells in *chinmo^ST^* testes. Interestingly, we also observed a progressive upregulation of a few genes associated with oocyte differentiation, such as *shavenbaby* (*ovo*) [1], *ovarian tumor* (*otu*) [1] and *Heterogeneous nuclear ribonucleoprotein at 27C* (*Hrb27C*) [16] over time in *chinmo^ST^* GSCs/spermatogonia (Fig. 3E and F). Notably, the expression levels of *ovo* and *otu* in *chinmo^ST^* GSCs/spermatogonia fall in between wild-type male and female germ cells (Fig. S9D and E). The 556 genes downregulated in *chinmo^ST^* GSCs/spermatogonia are enriched in mitochondrial translation and male gamete generation, suggesting that the cellular energetic flow is unbalanced, and that normal spermatogenesis may be transcriptionally restricted (Fig. 3E).

In *chinmo^ST^* early spermatocytes, genes such as male germline marker *PHD finger protein 7* (*Phf7*) [3,24] retain wild-type male expression (Fig. S9F), while 120 genes enriched in ribosomal activity and translation elongation are upregulated (Fig. S7H and I). We observed a progressive upregulation of a few genes involved in oocyte differentiation such as *eukaryotic translation initiation factor 4A* (*eIF4A*) [25] and *stubarista* (*sta*) [16] (Fig. 3F, Fig. S7G). Fifty-seven genes are downregulated in *chinmo^ST^* early spermatocytes and enriched in mRNA processing and spermatogenesis (Fig. S7H and J). Moreover, one of the testis-specific meiotic arrest complex core components and human LIN52 paralog *Wake-up-call* (*wuc*) [26] is downregulated in *chinmo^ST^* early germ cells (Fig. 3E and F). The absence of *wuc* expression may cause defects in the activation of gene expression in primary spermatocytes [26]. This is consistent with the *chinmo^ST^* phenotype exhibiting extensive proliferation of small cells resembling undifferentiated germ cells and a lack of mature germ cells [8,9,11].

Taken together, our results uncover the characteristics of mutant-specific GSCs/spermatogonia and early spermatocytes in *chinmo^ST^* testes. Although the surrounding CySCs exhibit feminization, the *chinmo^ST^* GSCs maintain their male-sex identity to a large extent and undergo overproliferation. On the other hand, the descendant mutant-specific spermatogonia-to-spermatocyte-transition cell population adopts a confused sexual fate and is unable to differentiate properly into late-stage germ cells (Fig. 3G).

### Soma-germline communication networks suggest that insulin signaling may contribute to the *chinmo^ST^* phenotype

Interactions between the soma and germline are essential throughout gametogenesis. Notably, approximately one-third of fly genes associated with male sterility in Flybase [27] show high expression specifically in somatic cyst cells of wild-type testes, with a significant enrichment in CySCs (Fig. S10A). This suggests the indispensable role of soma in germline development. To investigate how soma-germline interactions change as the feminization of somatic cyst cells in *chinmo^ST^* testes progresses, we next performed intercellular communication analysis across wild-type and *chinmo^ST^* testes.

To comprehensively characterize the interactions between somatic cyst cells and germ cells in testes, we first manually curated a comprehensive signaling molecule interaction database for *Drosophila* (Supplementary Table S2). It contains 256 molecular interactions, 86.7% of which are secreted signaling interactions, 12.1% are extracellular matrix-receptor interactions and 1.17% are cell–cell contact interactions (Fig. S10B). Intercellular communications among major cell types in *chinmo^ST^* and wild-type testes were inferred at three levels: ligand-receptor pair interaction level, signaling pathway level and the aggregated level (see ‘Methods’). We detected 5164 and 5324 significant ligand-receptor pairs among 11 major cell types in wild-type and *chinmo^ST^* testes, respectively (Supplementary Table S3). These signals were further categorized into 14 distinct pathways, including Bone Morphogenetic Protein (BMP), Activin, JAK-STAT, Epidermal Growth Factor Receptor (EGFR), Tumor Necrosis Factor (TNF), Hedgehog, Fibroblast Growth Factor Receptor (FGFR), Insulin-like Receptor (IR), Wnt-TCF, Wnt/Ca^2+^, Notch, Platelet-Derived Growth Factor/Vascular Endothelial Growth Factor (PDGF/VEGF)-receptor related (Pvr), Hippo and Toll signaling pathways. Known essential regulators of stem cell maintenance and niche function, such as BMP and Hedgehog signaling pathways [28,29], were well-recapitulated in the intercellular communication network of wild-type testes (Fig. S10C and D), indicating that our cell–cell communication analysis is capable of revealing signaling communication between cell types.

**Figure 4.**
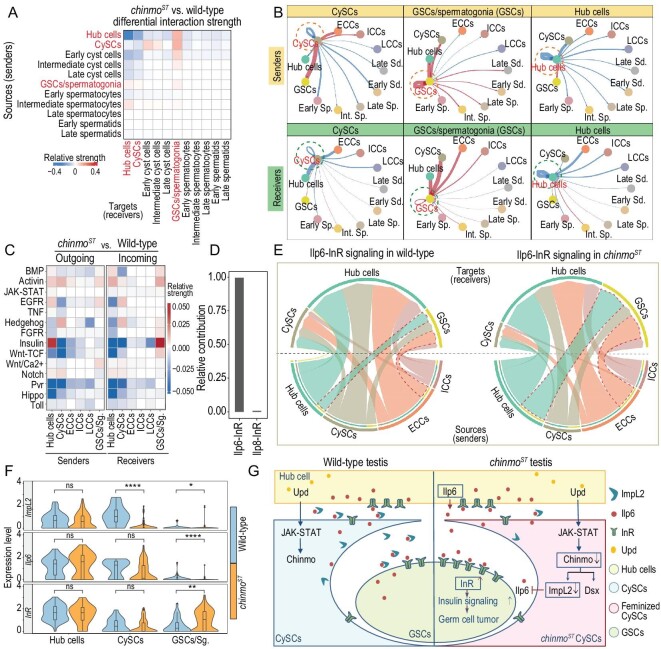
scRNA-seq reveals that the soma-germline communication network and insulin signaling are major players in stem cell sexual maintenance. (A) Heatmap showing differential interaction strength among each cell type in *chinmo^ST^* versus wild-type testes. Red and blue shading represents increased and decreased signaling strength in *chinmo^ST^* testes compared to wild-type testes, respectively. (B) Circle plots showing *chinmo^ST^* versus wild-type differential intercellular interaction strengths between CySCs (left), GSCs/spermatogonia (middle), hub cells (right) and other testis cell types. CySCs, GSCs/spermatogonia and hub cells are treated as signal senders in the upper three panels and signal receivers in the lower three panels. The dotted lines highlight signal senders/receivers. Widths of edge lines are proportional to the differential interaction strength. Red and blue edge lines indicate increased and decreased interaction strength in *chinmo^ST^* testes, respectively. (C) Heatmap showing differential outgoing and incoming signals sent or received by hub cells, somatic cyst cells and GSCs/spermatogonia in *chinmo^ST^* versus wild-type testes. Red and blue shading represents increased and decreased signaling strength in *chinmo^ST^* testes, respectively. (D) Bar plot showing relative contribution of each identified ligand-receptor pair to the overall insulin signaling targeted to *chinmo^ST^* GSCs/spermatogonia, which is the ratio of the communication probability of each ligand-receptor pair to that of insulin signaling targeted to *chinmo^ST^* GSCs/spermatogonia. (E) Chord diagram showing the communication probabilities mediated by ligand-receptor pair Ilp6-InR among hub cells, CySCs, GSCs/spermatogonia (GSCs), ECCs and ICCs, in wild-type and *chinmo^ST^* testes, respectively. Signal receivers are shown in the top half of the chord diagram while signal senders are shown in the bottom half. The inner thinner bar colors represent the target cell types that receive signals from the corresponding outer bar; the inner bar width is proportional to the signal strength received by the targets. Taking all the signals in the niche as a whole, there is a proportional decrease of Dilp6 output from *chinmo^ST^* CySCs and a proportional decrease of insulin signals received by *chinmo^ST^* hub cells and CySCs. It is possible that Chinmo-reduction induced feminization in CySCs downregulates the output of insulin-like ligand Dilp6. This could potentially trigger a compensatory increase in the output of Dilp6 from hub cells. However, most of the increased output of Dilp6 from hub cells is received by the mutant GSCs/spermatogonia, resulting in a distinct elevation of insulin signaling in these cells. (F) Violin plot showing the expression level of insulin pathway genes, including ligand *Ilp6*, receptor *InR* and ligand inhibitor *ImpL2* in hub cells,CySCs and GSCs/spermatogonia. Differential expression analysis is performed by Wilcoxon rank sum test (*, *P* < 0.05; **, *P* < 0.01; ****, *P* < 0.0001; ns, non-significant). (G) Working models of Chinmo-reduced CySCs activating insulin signaling in *chinmo^ST^* GSCs and thus initiating testis germ cell tumorigenesis. WT, wild-type. Sg, spermatogonia. Sp, spermatocytes. Sd, spermatids. Int, intermediate.

Next, we determined cell-type-specific signaling changes between *chinmo^ST^* and wild-type testes. Among all 11 cell populations, cells within the testis apex, including hub cells, CySCs and GSCs/spermatogonia, display dramatic changes in the outgoing and incoming interaction strength in *chinmo^ST^* testes (Fig. 4A, Fig. S10C and E). Thus, we then focused on these three cell types. We observed that *chinmo^ST^* CySCs show elevated outgoing signaling to the aggregates of sexually transformed early cyst cells and GSCs/spermatogonia, and reduced outgoing signaling to themselves and hub cells (Fig. 4A and B). In *chinmo^ST^* testes, GSCs/spermatogonia are found to be the ‘super receiver and sender’. They have an elevated potential for receiving and sending signals from most other major cell populations, with hub cells and CySCs being two major interactors (Fig. 4A and B). This is consistent with the requirement for cyst cells, which wrap GSCs/spermatogonia and their descendants to prevent germ cell overproliferation [8,9,11]. Hub cells show stronger interactions with GSCs/spermatogonia in *chinmo^ST^* testes (Fig. 4A and B). However, they show reduced interactions with themselves and the feminized CySCs (Fig. 4A and B). This suggests that hub cells may lose their ability to communicate with adjacent CySCs at the onset of CySCs feminization.

To identify the specific signaling pathways involved in CySC sex conversion and germ cell overproliferation, we further analyzed the 14 distinct pathways mentioned above (Fig. 4C). Four pathways are all significantly altered in *chinmo^ST^* CySCs: TGF-β (both BMP and Activin signaling), EGFR, insulin and Pvr (Fig. 4C). In *chinmo^ST^* GSCs/spermatogonia, the most prominent change was the upregulation of the IR signaling pathway (Fig. 4C), which homeostatically maintains and regulates testis and ovarian GSCs [30]. Furthermore, a previous study measuring somatic body size plasticity shows that the sex determination pathway governs a female-biased increase in insulin signaling activity [31]. We speculated that the insulin signals might contribute to somatic-sex-conversion-induced germ cell tumorigenesis.

Among all known ligand-receptor pairs, the insulin signaling pathway targeted to *chinmo^ST^* GSCs/spermatogonia was dominated by the *insulin-like peptide 6* (*Dilp6*) ligand and its receptor *insulin-like Receptor* (*InR*) (Fig. 4D). By further analyzing Dilp6-InR signaling in different cell types, we found that GSCs/spermatogonia received more IR signals mainly from hub cells and early cyst cells in *chinmo^ST^* testes (Fig. 4E). Consistent with the signaling changes, the receptor *InR*, downstream effectors of the IR signaling pathway such as *Akt kinase* (*Akt1*) and *Phosphatidylinositol 3-kinase 92E* (*Pi3K92E*) are significantly upregulated in *chinmo^ST^* GSC/spermatogonia over time (Fig. 4F, Figs S3E–G, S10F). Notably, their expression levels are non-sex-specific in wild-type testes and ovaries (Fig. S9G–I). This observation suggests that these factors are abnormally upregulated in *chinmo^ST^* germ cells, rather than adopting a female-specific expression pattern. Additionally, *ImpL2*, which binds the ligand Dilp6 to antagonize insulin signaling [32], is significantly downregulated in feminized CySCs (Fig. 4F). Loss of *ImpL2* could promote the short-range diffusion of Dilp6, further increasing insulin signaling in GSCs/spermatogonia as surrounding somatic cells become feminized [32].

Taken together, our results suggest that the intercellular communications in the *chinmo^ST^* testis niche are vastly changed, mainly through alterations in the insulin, TGF-β and EGFR signaling pathways. Aside from the canonical sex determination pathway, the insulin signaling pathway is presumed to explain the dramatic change in *chinmo^ST^* testis niche and could mediate the crosstalk between Chinmo-reduction induced feminization of CySCs and germ cell tumorigenesis, directly or indirectly (Fig. 4G).

### Insulin signaling is involved in stem cell differentiation and sex maintenance

**Figure 5.**
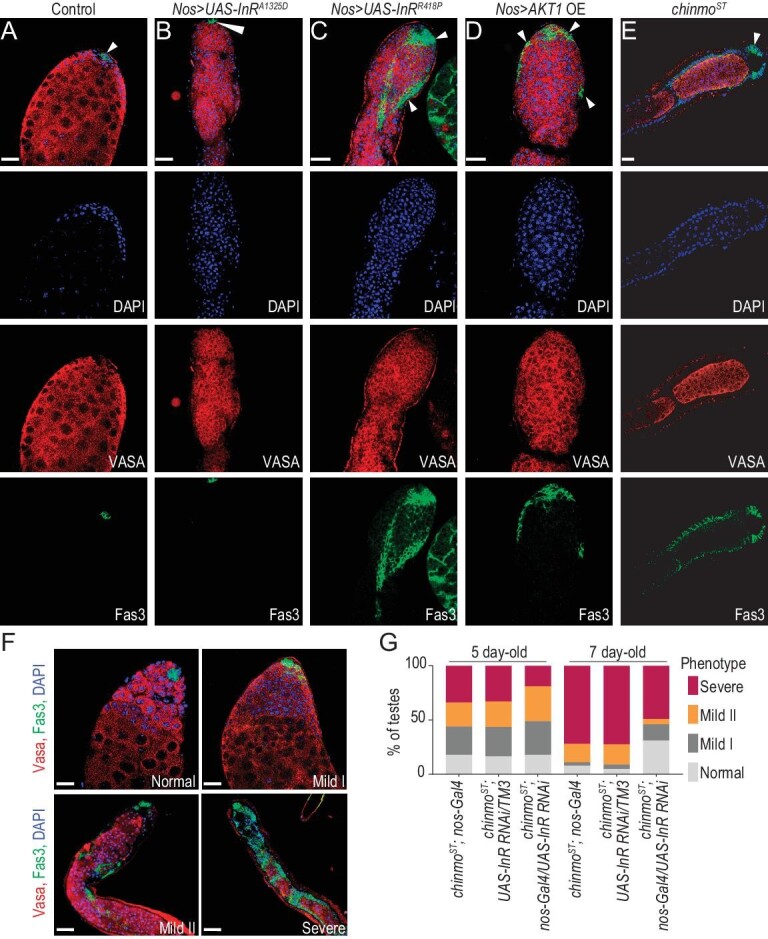
Insulin signaling components are involved in stem cell differentiation and sex maintenance. (A–E) Two-week-old whole adult testes immunostained for Vasa (germline), Fas3 (hub membranes) and DAPI (DNA) in control (*Nos-Gal4 > UAS-GFP*) (A), *Nos-Gal4 > UAS-InR^A1325D^* (expresses a constitutively active InR under the control of UAS; the A1325D amino acid change mimics the human V938D change) (B), *Nos-Gal4 > UAS-InR^R418P^* (expresses a constitutively active InR under the control of UAS; the R418P amino acid change mimics the human K86P change) (C), *Nos-Gal4 > UAS-Akt1* (D) and *chinmo*^*ST*^ (E). Arrowheads mark somatic cyst cells. (F) The follicle-like cell phenotype in 2-week-old *chinmo^ST^* testes (E) can be partially rescued by knocking down InR in the *chinmo^ST^* GSC lineage (*chinmo^ST^; Nos-Gal4/UAS-InR RNAi*), displaying a range of mild to severe phenotypes [11]. (G) Composite bar graph showing the percentage of testes with normal, mild-I, mild-II and severe phenotypes after knocking down InR in adult *chinmo^ST^* GSC lineage (*chinmo^ST^; Nos-Gal4/UAS-InR RNAi*) for the number of days indicated. Control genotypes consist of *chinmo^ST^; Nos-Gal4* and *chinmo^ST^; UAS-InR RNAi*/*TM3*. Morphologically normal wild-type testes were scored as ‘normal’. Testes with Fas3-positive epithelial aggregates at the apex near the hub were scored as ‘mild-I’; these testes often contained overproliferating germ cells. Testes displaying Fas3-positive epithelial aggregates and overproliferating early germ cells approximately halfway from the distal end were classified as ‘mild-II’. Testes with Fas3-positive epithelial aggregates at the distal 2/3 or throughout the entire testis were scored as ‘severe’; these testes often showed germ cells enclosed by these epithelial aggregates, leading to their arrest at an early spermatogonial stage, or to degeneration [11]. Scale bars = 20 μm.

Our single-cell data suggested that increased insulin signaling received by germ cells shifts the fate of *chinmo^ST^* GSCs toward tumorigenesis. To test this hypothesis, we first knocked down the insulin ligand antagonist *ImpL2* within CySCs, since *ImpL2* expression decreases in CySCs undergoing feminization. This yielded testes indistinguishable from wild-type, consistent with a previous report [32]. This shows that decreased insulin ligand antagonist *ImpL2* alone is insufficient to produce germ cell phenotypes. Since the *insulin-like receptor* (*InR*) is upregulated in mutant GSCs/spermatogonia, we next overexpressed *InR* in GSCs. This resulted in the formation of aggregates of Fas3-positive somatic cells along with the presence of DAPI-bright overproliferating germ cells (38%–67% of testes; *n* = 294), similar to what we observed in *chinmo^ST^* testes (Fig. 5A–C, E, Supplementary Tables S4 and S5). Notably, the formation of the follicle cell-like epithelial aggregates presents a milder phenotype with relatively lower penetrance compared to fully developed columnar follicle cell-like epithelia throughout the testes observed in *chinmo^ST^* testes (∼95% of testes) [11] (Fig. 1A′′′′, Supplementary Tables S4 and S5). In addition, we overexpressed a downstream component of the insulin pathway (*Akt1*) in germ cells; this also resulted in somatic aggregates and overproliferating early germ cells (44%–48% of testes; *n* = 82), consistent with the above observations (Fig. 5A, D and E, Supplementary Tables S4 and S5). We further hypothesized that if *chinmo^ST^* testes acquire phenotypes as a result of increased insulin signaling in germ cells, we could potentially reverse these phenotypes by decreasing insulin signaling in germ cells of *chinmo^ST^* testis. Knockdown of InR in GSCs using the driver *nanos-Gal4* in the *chinmo^ST^* background partially rescued phenotype progression (one-week-old phenotype penetrance decreased from 90% to 70%) (Fig. 5F and G, Supplementary Tables S6 and S7). This indicates that the feminization of CySCs can induce GSC fate toward tumorigenesis via the insulin signaling pathway. However, the insulin signaling may not be the only pathway involved since it cannot fully rescue the *chinmo^ST^* mutant phenotype.

Taken together, our results imply that Chinmo is working through both the canonical sex determination pathway and the insulin signaling pathway to maintain the male identity of CySCs and the integrity of germ cell differentiation. Sex conversion of CySCs in *chinmo^ST^* testes disrupts soma-germline communication through the insulin pathway and leads to differentiation defects.

### Chinmo directly targets and activates *dsx* and insulin pathway members

**Figure 6.**
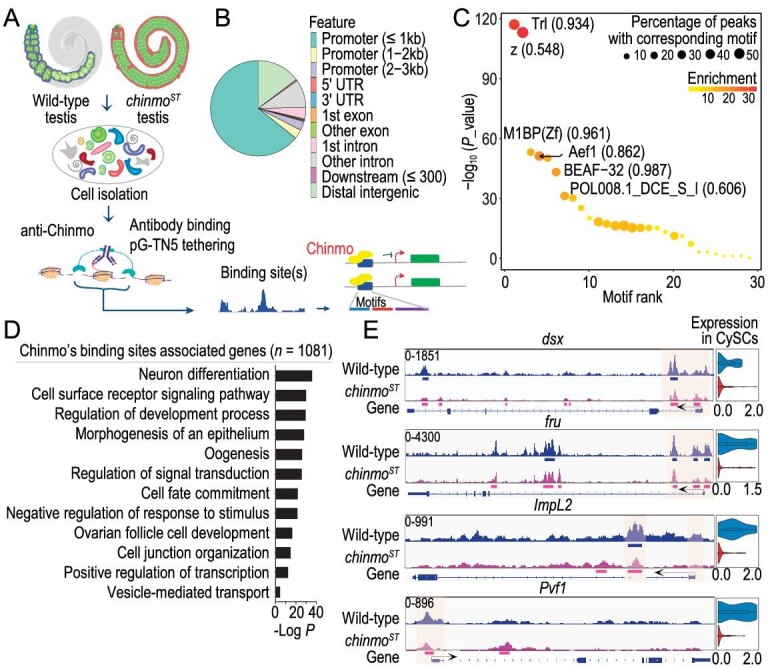
Chinmo directly targets and activates *dsx* and insulin pathway members. (A) Workflow for CUT&Tag sequencing using anti-Chinmo antisera. (B) Pie chart showing the percentage of reproducible Chinmo binding sites in wild-type testis that fall on different genomic regions. (C) Dot plot showing enriched motifs of Chinmo binding sites in wild-type testis. Dot size represents percentage of target peaks with corresponding motif and dot color represents degree of motif enrichment in target peaks compared to random background sequences. Enriched motifs that are found in >15% of target peaks are labeled with the most similar known motifs and similarity scores. The x-axis represents rank of motif ordered by *P* value. (D) Top GO terms enriched for Chinmo peaks associated genes (*n* = 1081) in wild-type testis. (E) Genome tracks for exemplary Chinmo direct targets identified by CUT&Tag, including *dsx, fru, ImpL2* and *Pvf1* in wild-type and *chinmo^ST^* testis. Violin plots on the right showing expression of these genes in wild-type and *chinmo^ST^* testis, respectively. The colored blocks beneath Chinmo peaks represent MACS2-called peaks, which are considered genuine Chinmo binding sites in wild-type and *chinmo^ST^* testis, respectively. WT, wild-type. Exp, expression.

Although Chinmo, as a Zinc finger C2H2 protein, is reported as a putative transcription factor [9,33,34], no direct binding targets for Chinmo have been identified so far. We applied CUT&Tag assay to probe Chinmo binding sites (Chinmo peaks) in wild-type and *chinmo^ST^* testes (Fig. 6A). After rigorous quality control (Fig. S11A–D and see ‘Methods’), we obtained 1290 and 3425 peaks in wild-type and *chinmo^ST^* testes, respectively (Fig. S11E). Most Chinmo binding sites in testes were distributed within promoter regions (≤1 kb) (Fig. 6B). Enriched motifs of these sites shared high similarity with known motifs including Trithorax-like (Trl), Motif 1 Binding Protein (M1BP), Adult enhancer factor 1 (Aef1) and Boundary element-associated factor of 32 kD (BEAF-32) (Fig. 6C). Wild-type Chinmo peaks with these motifs have distinct protein binding footprints (Fig. S11F), suggesting that Chinmo might interact or share similar motifs with these factors. 1081 genes associated with Chinmo binding sites are enriched in the regulation of epithelial development, neurogenesis, cell communication, female gamete generation and cell fate commitment (Fig. 6D), which is consistent with the identity of Chinmo as a neuronal temporal regulator, sex determination effector and stem cell maintenance factor [8–11,34–36]. Compared to Chinmo motif-containing sites identified previously through bacterial one-hybrid approaches [37], our data showed 507 overlapping Chinmo peaks. This included several targets previously predicted as putative targets of Chinmo (such as *mirror* (*mirr*), *scribble* (*scrib*), *polychaetoid* (*pyd*) and *DE-cadherin* (*shg*)) [8], identified through recognition of a Chinmo motif using the bacterial one-hybrid system (Fig. S12).

We further asked which genes are directly targeted by Chinmo in feminized CySCs by performing differential binding sites (DBSs) analysis between *chinmo^ST^* and wild-type testes. 1426 upregulated and 1947 downregulated Chinmo-binding sites were identified in *chinmo^ST^* testes. Since Chinmo is depleted in the CySC lineage and slightly upregulated in GSCs/spermatogonia and early germ cells in *chinmo^ST^* testes (Fig. 1D and E, Fig. S3B and D), we assumed that the downregulated DBSs represent Chinmo-binding sites in CySCs. By overlapping the associated genes of downregulated Chinmo-binding sites and DEGs in *chinmo^ST^* CySCs, we identified 152 direct targets of Chinmo in CySCs (Supplementary Table S8). These target genes are enriched in cell fate commitment, ovarian follicle cell development and sex differentiation (Fig. S11G). These results corroborate the above-mentioned findings that Chinmo is a major contributor influencing the sex maintenance and fate commitment of CySC lineage cells.

Among 152 direct targets of Chinmo in CySCs, we found that some canonical sex determination factors and factors of the above-mentioned differential signaling pathways are top-ranked (Fig. 6E). Previously we showed that Chinmo maintains the male identity of CySCs through the canonical male determination factor Dsx [11]. Here we verified that Chinmo is directly activating *dsx* as well as another male sex determination factor *fru* in CySCs (Fig. 6E). Furthermore, the negative regulator of insulin signaling *ImpL2* and the Pvr signaling ligand *PDGF- and VEGF-related factor 1* (*Pvf1*), members of differentially signaling pathways between *chinmo^ST^* and wild-type testes, are also directly activated by Chinmo in CySCs (Fig. 6E). These data suggest that Chinmo can directly target and activate the expression of sex determination factors and insulin pathway components to regulate sexual maintenance in CySCs. Additionally, genes upregulated in mutant GSCs/spermatogonia, such as the female germline sex determination factors *ovo* and *otu* [1], also display Chinmo binding and this binding increases in *chinmo^ST^* testes (Fig. S13A). However, we have also uncovered Chinmo's role as a transcriptional repressor for some genes (Fig. S13B). Upon comprehensive analysis of Chinmo binding and its targets’ expression, our results suggest that in both soma and germline, Chinmo predominantly functions as a transcriptional activator to promote the expression of male-biased genes (Fig. S13C and D).

## DISCUSSION

The *chinmo^ST^* testis serve as an excellent model for investigating the molecular mechanisms underlying sex maintenance of somatic CySCs and the associated intercellular signaling between somatic cells and germ cells. Through scRNA-seq and the Chinmo CUT&Tag assay on both wild-type and mutant testes, we found that the reduction of Chinmo in *chinmo^ST^* CySCs leads to the downregulation of *dsx^M^* and the upregulation of FSC markers at the transcription level, thereby triggering a comprehensive transcriptional response to promote feminization. Furthermore, our data revealed significant disruptions in various intercellular communication pathways within the *chinmo^ST^* testes, with the insulin-receptor signaling pathway exhibiting the most pronounced alterations. Remarkably, some major components of the insulin-receptor signaling pathway are directly bound by Chinmo. In combination with phenotypic verification via genetic manipulation in CySCs and GSCs, we validated that the insulin pathway signaling components, including the insulin receptor and its downstream effector *Atk1*, are major contributors to germline defects seen in *chinmo^ST^* testes. Altogether, our work indicates that Chinmo directly regulates the male sex determination factor *dsx*, the fly homolog of the mammalian *DMRT1*, as well as other intercellular signals such as insulin signaling, to maintain male identity of the CySC lineage cells and promote normal spermatogenesis.

The mechanism of sex determination in *Drosophila* is orchestrated by the cascade of sex-specific splicing of mRNA. Our study reveals that the molecular mechanisms underlying sex maintenance in adult files involve canonical sex determination genes. Specifically, Chinmo, as a key factor to maintain the male identity of adult CySCs, can directly regulate the transcriptional levels of sex determination genes including *dsx* and *fru*. Furthermore, Chinmo has also been shown to repress alternative splicing of *tra* pre-mRNA to maintain male sex identity of CySCs [9]. Our CUT&Tag data reveal that Chinmo binds to the promoters of splicing factor genes like *U2 small nuclear riboprotein auxiliary factor 50* (*U2af50*), which is a *Sex lethal* (*Sxl*) [3] antagonist that can regulate alternative splicing of *tra* pre-mRNA [38]. Hence, Chinmo potentially regulates both gene expression and alternative splicing. However, our scRNA-seq data only capture the 3ʹ end of genes, which limits our ability to detect sex-specific splicing in canonical sex determination genes. Further work is required to decipher the role of alternative splicing in sex maintenance.

In this study, we report the cell–cell communication networks in the *Drosophila* testis for the first time, and offer insights into additional functional signaling pathways. By comparing intercellular communications between wild-type and *chinmo^ST^* testes, we found significant changes in several signaling pathways, particularly the insulin signaling pathway, which accompanies the *chinmo^ST^*-mediated sex transformation and defective gametogenesis. Furthermore, soma-germline signaling pathways such as the TGF-β pathway (BMP and Activin signaling), EGFR and PVR signaling were found to be activated or repressed in mutant CySCs (Fig. 4C). A previous study has shown that ectopically activated Activin signaling in GSCs results in overproliferation of both stem-cell-like and spermatogonial-like cells [39], which is consistent with the *chinmo^ST^* phenotype. This indicates that in addition to the insulin signaling, other detected signaling changes may also contribute to the disrupted gametogenesis. It also helps to explain why a sole decrease of insulin ligand antagonist *ImpL2* in CySCs is insufficient to phenocopy germ cell phenotypes. It is plausible that the reduction of Chinmo in CySCs promotes the expression of *InR* in GSCs through any of these soma-germline signaling pathways, thus priming for increased insulin signaling in *chinmo^ST^* GSCs. Further investigations are required to unravel the contribution of other membrane-mediated signal exchanges and dysregulated genes in this process.

Whether germline sex transformations are true within each cell or instead represent mosaic intersexes is a long-standing question. Previous methods relied primarily on immunostaining for a limited number of markers to assess the sex status of cells [1,3,11,19,21,40], or bulk sequencing of the entire tissue or sorted cells [8,9,24,41]. However, these methods pose challenges in determining the extent of sex conversion in the whole transcriptomic level within individual cell types. Our research, employing single-cell transcriptomic analysis, provides a comprehensive and high-resolution approach to address this question. Our investigation reveals a distinctive and partial transformation, as well as a degree of ambiguity in the sexual identity of germ cells in *chinmo^ST^*, supported by multiple lines of evidence. Firstly, our correlation analysis comparing mutant germ cells with both male and female counterparts indicates a decreased correlation with wild-type male cells and an increased correlation with female cells, yet this correlation remains partial (Fig. 3C, Fig. S7D). Furthermore, as the level of feminization changes over time, germ cells remain intermediate between male and female (Fig. S7C). Moreover, the sex conversion varies across developmental stages: *chinmo^ST^* GSCs/spermatogonia display closer resemblance to wild-type male counterparts, while *chinmo^ST^* early spermatocytes exhibit greater similarities with ovarian germline cells (Fig. 3C, Fig. S7D). Notably, *chinmo^ST^* early spermatocytes display distinctive within-population heterogeneity, consisting of cells in various states of sex-transformation (Fig. S8A and B). Examining differential gene expression patterns reveals a complex scenario: certain pathways, such as Hedgehog pathway components, maintain their original sex identity. Conversely, the expression levels of some Wnt pathway constituents fall into an intermediate range between male and female. Notably, male germline markers like *Pif1a* [13,22] and *Phf7* [3,24] maintain a predominantly male expression pattern (Fig. S9A and F). However, the expression levels of genes associated with oocyte differentiation, such as *ovo* and *otu* [1] lie in between wild-type male and female (Fig. S9D and E). These observations collectively underscore the intricate nature of partial sex transformations within the germline in *chinmo* mutant flies.

Since Chinmo plays a pivotal role in the manifestation of mutant phenotype, it is important to identify target genes and the regulatory nature of Chinmo. Although previous studies have proposed Chinmo as a putative transcriptional repressor [8,9,33,34], our findings illustrate its capacity to both activate and repress target gene expression (Fig. 6E, Fig. S13B). However, our observations lean towards a predominant activation of genes linked to cell fate commitment, ovarian follicle cell development and sex differentiation within *Drosophila* testis (Fig. 6D, Fig. S13C and D). Interestingly, the enriched motifs identified in Chinmo binding sites exhibit notable similarity to known motifs such as Trl, Aef1 and BEAF-32, which have been reported to function as either transcriptional activators or repressors [42,43]. Chinmo might interact with distinct cofactors to modulate its regulatory functions. Future work is needed to determine the intricate regulatory nature of Chinmo across different cell types. Additionally, the observed increase of Chinmo binding peaks in *chinmo^ST^* testes may be attributed to elevated Chinmo expression in germ cells within *chinmo^ST^* testes (Fig. 1D, Fig. S3D). However, without cell-type-specific CUT&Tag data, it is not possible to definitively confirm whether these Chinmo-binding peaks are exclusively located in germ cells.

Chinmo has structurally conserved orthologs extending to the Palaeoptera [44], which can be dated back to 400 million years ago. However, the extent of its functional conservation remains unclear. Exploring whether Chinmo homologs play a role in sex maintenance in other organisms is an intriguing area for future investigation. Beyond the functional conservation of Chinmo protein, our findings reveal that disturbances in somatic sex maintenance disrupt soma-germline communication, thus leading to impaired gonadal gametogenesis. This provides valuable insights into human testicular diseases observed in adult males. (Stem) cell sex confusion or conversion might be a potential mechanism contributing to defective gametogenesis shared among flies, mice and humans.

## MATERIALS AND METHODS

A detailed description is included in Supplementary Materials and Methods.

## Supplementary Material

nwae215_Supplemental_files

## Data Availability

scRNA-seq and CUT&Tag data have been deposited at NCBI Gene Expression Omnibus (GEO) repository, with accession numbers GSE201673 (reviewer token: ilitqseofvaxhuz) and GSE201579 (reviewer token: olmjkgqobvaptex), respectively. Chinmo CUT&Tag data sets are viewable on the University of California Santa Cruz (UCSC) Genome Browser (http://genome.ucsc.edu/s/PeggySze/Fly_testis_Chinmo_CUT&Tag). All original codes are provided at the GitHub repository (https://github.com/PeggySze/ChinmoST). All other data are available in the main text or the supplementary files.
