# Supplementary material for: Soma-germline communication drives sex maintenance in the Drosophila testis
Source: Natl Sci Rev. 2024 Jun 22;11(8):nwae215. doi: 10.1093/nsr/nwae215 (PMC11342250; doi:10.1093/nsr/nwae215)
Supplement: nwae215_Supplemental_files [file nwae215_supplemental_files.zip › NSR_MS-2024-601.R1 Supplemental_files/[NSR_MS-2024-601.R1] Supplemental file.docx]

**Supplementary Information for**

**Soma-Germline Communication Drives Sex Maintenance in the *Drosophila* Testis**

Rui Zhang,^1#^ Peiyu Shi,^2#^ Shuyang Xu,^1^ Zhe Ming,^1^ Zicong Liu,^1^ Yuanyuan He,^1^ Junbiao Dai,^1^ Erika Matunis,^3^ Jin Xu,^2*^ Qing Ma^1*^

***Correspondence**: Qing Ma (qing.ma@siat.ac.cn) and Jin Xu (xujin7@mail.sysu.edu.cn)

**Email:**  [qing.ma@siat.ac.cn](mailto:qing.ma@siat.ac.cn), [xujin7@mail.sysu.edu.cn](mailto:xujin7@mail.sysu.edu.cn)

**This PDF file includes:**

Supplementary Materials and Methods

Supplementary Figures S1 to S13

Legends for supplemental Table S1 to S3

Supplemental Tables S4 to S7

Legends for supplemental Table S8

SI References

Supplementary Materials and Methods

**Fly Stocks**

Fly stocks were raised at 25 °C on standard molasses/yeast medium unless otherwise indicated. We used *traffic jam* (*tj*)*-Gal4* (Drosophila Genetic Resource Center), c587-Gal4[1], *y w;;nos-gal4* (TsingHua Fly Center, TB00145), *upd-Gal4* (TsingHua Fly Center, THJ0197), *bam-Gal4* (gift from T. Guo), *UAS-GFP^nls^* (Bloomington Drosophila Stock Center, BDSC-4775), *UAS-chinmo^HMS00036^ -RNAi* (BDSC-33638), *chinmo^ST^* [2], *UAS-ImpL2 RNAi* (TH03104.N), *UAS-InR^HMS03166^ RNAi* (THU5741), *UAS-ilp6^HMS00549^ RNAi* (THU0985), *UAS-Pvf1^HMS01958^ RNAi* (THU3954), *UAS-InR^R418P^* (BDSC-8250), *UAS-InR^A1325D^* (BDSC-8263) and *UAS-Akt1*(III) (Core Facility of Drosophila Resource and Technology, SIBCB, CAS. BCF241). *Oregon R* flies were used as control flies in single-cell RNA-seq. Other fly stocks were from the BDSC, TsingHua Fly Center and Core Facility of Drosophila Resource and Technology, SIBCB, CAS.

**Immunostaining**

Testes and ovaries were dissected, fixed, and stained as described previously[3]. Antibodies used for immunostaining included: rat anti-Vasa (1:250, DSHB), mouse anti-Fas3 (1:800, DSHB), rat-anti-Chinmo[4] (1:500; a gift from Dr. Sokol), rabbit anti-Chinmo (1:5000; this study against a His-tagged version of full-length Chinmo-PA protein[4]), rabbit anti-Tj[5] (1:1000, a gift from Dr. Wang), mouse anti-Armadillo (1:50, DSHB) and chicken anti-GFP (1:2000, ab13970) . Samples whose staining was directly compared were prepared and imaged in parallel and under identical conditions.

**Single-cell RNA-seq**

*chinmo^ST^* and wild-type males at 3–5, 6–8, and 9–11 day-old were collected, and approximately 100 pairs of testes were dissected for each sample preparation. Testes were dissected into Schneider’s medium and then washed twice with cold 0.04% BSA DPBS solution. Five hundred microliters 0.25% Trypsin was added to each tube/sample. Samples were vortexed with 1000 rpm for 15-18 minutes at room temperature. The enzymatic reactions were terminated by adding 500 µL DMEM (with >10% FBS). Cell suspensions were filtered through a 40 µm Falcon filter into an Eppendorf tube. Spermatids were filtered out for further enrichment of early germ cells and somatic cyst cells. The efficiency of single cell dissociation methods for fly testes were tested by labelling somatic cells or germline cells with GFP fluorescence. The percentage of each type of GFP-labelled cells were measured, ensuring retrieval of single cell suspensions with a reasonable amount of viable germline and somatic cells (Figure S1B).

Single-cell RNA-seq libraries were prepared using the Chromium Single Cell 3ʹ Reagent Kits v3 (10x Genomics), according to the manufacturer’s instructions. Briefly, approximately 5,000–10,000 cells were washed with 0.04% BSA DPBS for three times and were resuscitated and manually determined to a concentration of 700~1200 cells/μL (viability≥85%). Cells were captured in droplets at a targeted cell recovery of cells. After the reverse transcription step, emulsions were broken and Barcoded-cDNA was purified with Dynabeads, followed by PCR amplification. Amplified cDNA was then used for 3’gene expression library construction. For gene expression library construction, 50 ng of amplified cDNA was fragmented and end-repaired, double-size selected with SPRIselect beads, and sequenced on the NovaSeq platform (Illumina) to generate 150 bp paired-end reads.

**CUT&Tag library generation and sequencing**

5–7 and 9–11 day old wild-type and *chinmo^ST^* males were collected, and approximately 100 pairs of testes were dissected for each sample preparation. Testes dissection and single cell dissociation were as described above in single-cell RNA-seq. CUT & Tag was performed as previously described [6] with modifications. The NovoNGS ® CUT & Tag 2.0 High-Sensitivity Kit (Vazyme, Nanjing, China, N259-YH01) was used to capture Chinmo-binding sites.

Binding buffer was prepared by mixing 200 μL 1 M HEPES pH 7.9, 100 μL 1 M KCl, 10 μL 1 M CaCl2 and 10 μL 1 M MnCl2, and dH2O into a 10 mL cocktail. Wash buffer was prepared by mixing 1 mL 1 M HEPES pH 7.5, 1.5 mL 5 M NaCl, 12.5 μL 2 M spermidine, 1 Roche Complete Protease Inhibitor EDTA-Free tablet and dH2O into a 50 mL cocktail. Dig-wash buffer was prepared by mixing 400 μL 5% digitonin with 40 mL Wash buffer. Antibody buffer was prepared by mixing 8 μL 0.5 M EDTA and 6.7 µL 30% BSA with 2 mL Dig-wash buffer. Dig-300 buffer was prepared by mixing 1 mL 1 M HEPES pH 7.5, 3 mL 5 M NaCl and 12.5 μL 2 M spermidine, 100 µL 5% digitonin (0.01%), one Roche Complete Protease Inhibitor EDTA-Free tablet and dH2O into a 50 mL cocktail. Tagmentation buffer was prepared by mixing 5 mL Dig-300 buffer and 50 µL 1 M MgCl2.

Briefly, cells were incubated with 10 μL pre-washed ConA beads in a 1.5-mL low-binding tube. Fifty microliters of antibody buffer with 1 μg antibody (rabbit anti-Chinmo-PA, 1:50) was added and cultured overnight at 4°C. After washing twice with dig-wash buffer, 50 μL dig-wash buffer with 0.5 μg secondary antibody was added and incubated at room temperature for 1 h. After washing twice with 800 μL dig-wash buffer, 1.0 μM pG–Tn5 was added with 100 μL Dig-300 buffer. Samples were incubated at room temperature for 1 h and then washed twice with 800 μL dig-wash buffer. Three hundred microliters of tagmentation buffer were added and samples were incubated at 37 °C for 1 h. The reaction was stopped with 10 μL 0.5 M EDTA, 3 μL 10% SDS and 2.5 μL 20 mg/mL Proteinase K. After extraction with phenol-chloroform and ethanol precipitation, PCR was performed to amplify the libraries according to the manufacturer’s instructions. DNA quality and quantity were determined using the Agilent 2100 Bioanalyzer. Post-PCR clean-up was performed by adding 1.3× volume of NovoNGS ^®^ DNA Clean beads (Vazyme, Nanjing, China, N259-YH01), according to the manufacturer's instructions. All libraries were sequenced by Illumina NovaSeq S4 to generate 150 bp paired-end reads.

**Single-cell RNA-seq processing and cell type identification**

For each scRNA-seq dataset, raw data were aligned to *D. melanogaster* reference genome dm6 and gene-barcode matrices were generated by CellRanger [7] version 4.0.0. Count data were further processed using the R package Seurat [8] version 3.2.2. High-quality cells that expressed 200–6,000 genes and had mitochondrial content <10% were retained for the downstream analysis. Single-cell RNA-seq datasets of wild-type and mutant testes across different time points were integrated using the standard integration workflow (https://satijalab.org/seurat/v3.0/integration.html). Briefly, feature counts from each sample were log-normalized using the NormalizeData() function and top 2000 variable features were identified using FindVariableFeatures(). To integrate scRNA-seq datasets, a set of anchors was identified using the FindIntegrationAnchors() with 2000 genes. These anchors were used to integrate six scRNA-seq datasets. Data were scaled using the ScaleData() function. To reduce dimensionality of integrated data, principal component analysis was performed. The top 30 principal components were selected based on the results from the JackStraw procedure and elbow plot and further used for clustering at a resolution of 0.4 and running the Uniform Manifold Approximation and Projection (UMAP) dimensional reduction. This resulted in four peripheral clusters and two cell groups which consist of somatic lineage and germline lineage in testes. Cells associated with the testis sheath (muscle, pigment) or that were inadvertenly captured along with testis cells, e.g. hemocytes, were first annotated using the following marker genes: Hemocytes (*Ppn*, *Hml*); Muscle cells (*Mp20*, *Zasp66*); Pigment cells (*glob1*, *CG2233*); Male gonad associated epithelium (*CG18628*, *MtnA*) (Figure 1B-C). To annotate somatic cells and germline cells in detail, somatic cells and germline cells were subset for subclustering analysis respectively [9-12]. Standard integration workflow of Seurat was applied to both somatic cells and germline cells subclustering analysis as mentioned above except for the number of chosen principal components and clustering resolution. The top 20 principal components were used for clustering at a resolution of 0.4 in germline cells subclustering analysis, while the top 30 principal components were used for clustering at a resolution of 0.6 in somatic cells subclustering analysis. Germline cells were annotated to six cell types (markers are listed): GSCs/spermatogonia (*aub, zpg, vas, nos, stg, bam*); Early spermatocytes (*Rbp4, kmg, wuc, nht*, *Taf12L*); Intermediate spermatocytes (*nht*, *Taf12L*, *sa*, *CycB*, *fzo*, *twe*, *CG3927*); Late spermatocytes (*CycB*, *fzo*, *CG3927*, *bol*); Early spermatids (*bol*, *Dpy-30L2*); Late spermatids (*schuy*, *hale*, *boly*, *whip*, *p-cup*, *wa-cup*, *r-cup*, *d-cup*) (Figure 1B-C, S2F-J). Somatic cells subclustering analysis enabled us to annotate the following cell types (markers[10] are listed): Early cyst cells (*Wnt4*, *CG31676*); Intermediate cyst cells (*Pdcd4*, *sog*, *Six4*); Late cyst cells (*CG8665*, *CG3376*); Terminal epithelial cells (*Nep2*, *Nep4*, *mnd*); Somatic cells at base of testis (*Ubx*) (Figure 1B-C, S2A-E). Due to the incapability to distinguish between hub cells and CySCs based on the above somatic cells subclustering analysis, hub cells, CySCs, early cyst cells and intermediate cyst cells were subset for further subclustering analysis. Standard integration workflow of Seurat was applied to cyst cells subclustering analysis with 1000 anchor features and the top 20 principal components for clustering at a resolution of 0.8. Hub cells and CySCs were annotated using the following marker genes [10]: Hub cells (*org-1*, *Fas3*, *InR*, *aop*, *Socs36E*); CySCs (*zfh1*, *ptc*, *piwi*, *tj*, *puc*, *stg*, *InR*, *aop*, *Socs36E*). Marker genes and differentially expressed genes between mutant and wild-type for each cell type were identified with the Wilcoxon rank-sum test by FindAllMarkers() function. Only those with fold change ≥ 1.5 and p-value < 0.05 were considered as marker genes or differentially expressed genes. Functional enrichment of differentially expressed genes were performed by clusterProfiler [13] version 3.14.3. and KEGGREST version 1.26.1[14].

**Correlation of pseudobulk** **transcriptomes**

Pseudobulk transcriptome of a cell group was calculated by the following steps: first, we normalized count data of scRNA-seq by Transcripts Per Million reads (TPM). Next, we calculated average normalized counts of all cells in a cell group for each gene. We regarded the gene-level average normalized counts as pseudobulk transcriptome. When computing correlation of two pseudobulk transcriptomes, shared genes were retained and Pearson correlation coefficients were used.

**Trajectory analyses (including RNA velocity and conventional trajectory inference analysis) and comparison across mutant and wild-type germline cells**

**RNA velocity analysis of germline cells**

With BAM files processed by Cellranger and genome annotation file, spliced/unspliced expression matrices were generated by velocyto[15] version 0.17.17 in a command line interface. These matrices were loaded into R and integrated into Seurat objects. GSCs/spermatogonia, early spermatocytes, intermediated and late spermatocytes of wild-type and mutant testes were subset for running RNA velocity estimation respectively with the following parameters: deltaT = 1, kCells = 10, fit.quantile = 0.02. RNA velocities were visualized on UMAP embedding with the following parameters: arrow.scale = 2, show.grid.flow = TRUE, min.grid.cell.mass = 0.5, grid.n = 40.

**Trajectory inference and comparison across mutant and wild-type germline cells**

First, single-cell RNA-seq data of GSCs/spermatogonia, early spermatocytes, intermediate and late spermatocytes were subset for trajectory inference. We used condiments[16] (https://hectorrdb.github.io/project/condiments/) to infer trajectories and assess differential gene expression patterns along pseudotime between mutant and wild-type germline cells. Using the sample type labels and UMAP embedding coordinates from Seurat, imbalance scores that measure the imbalance between the local and global distributions of sample type labels were calculated (Figure S7F) and the *topologyTest* was performed. Based on the significant differential topology (p-value < 2.2e-16), we fitted an individual trajectory for mutant and wild-type germline cells, respectively (Figure S7F). Due to unchanged skeleton structure of trajectories, two trajectories of mutant and wild-type germline cells were mapped to each other. With the mapped trajectories, gene expression was modeled with the negative binomial generalized additive model using 8 knots and differential gene expression analysis was performed by the *conditionTest* to identify differential expressed genes (Fold change > 1, FDR < 0.05) along pseudotime between mutant and wild-type.

**Intercellular communication analysis**

To construct cell-cell communication networks for wild-type and mutant testes, we first manually curated a comprehensive signaling molecule interaction database for *Drosophila* based on information from FlyBase, the Interactive Fly, KEGG and FlyPhone [17]. Next, these signaling molecule interaction information and scRNA-seq data with major cell types (hub cells, CySCs, early cyst cells, intermediate cyst cells, late cyst cells, GSCs/spermatogonia, early spermatocytes, intermediate spermatocytes, late spermatocytes, early spermatids, late spermatids and hemocytes) served as input for CellChat [18] package to infer intercellular communications in wild-type and mutant testes, respectively. At ligand-receptor pair interaction level, CellChat first identified differentially over-expressed ligands and receptors for each cell group. Intercellular communication probability value was calculated by the law of mass action based on the average expression values of a ligand by one cell group and that of a receptor by another cell group, as well as their cofactors (average expression are calculated after trimming 10% of the smallest values). Significant interactions were identified using a permutation test by randomly permuting the group labels of cells and then recalculating the interaction probability.

Specifically, the likelihood of cellular communication is mainly calculated by an equation[19]. For the complex of the receptor ligand, as long as the expression level of one of the subunits is 0, it is considered that it has no effect on the receptor ligand pair. The result of the equation is a three-dimensional matrix, where K x K represents the relationship between cell populations, and the third dimension N represents the likelihood of cell communication for a particular receptor ligand pair. For each value in the three-dimensional matrix, it can be understood as the possibility of communication between specific receptor ligand pairs among cell populations. The likelihood of the sender (score) is obtained by accumulating the likelihood of cellular communication of all exocrine ligands. The likelihood of a receiver (score) is obtained by accumulating the likelihood of all ligand-stimulated cellular communication modes. For each signaling pathway, there are multiple pairs of receptor ligand pairs in the communication network. The communication possibilities of all sender cell groups are obtained by adding up the cellular communication possibilities calculated above (3D matrix) along the sender cells (in the receptor ligand pair, the ligand-secreting cell population) to obtain the communication possibilities of all sender cell groups (2D matrix, focusing only on all possibilities of ligand-secreting cell populations, not receiver cells). At a signaling pathway level, CellChat summarized the communication probabilities of all ligands-receptors interactions associated with each signaling pathway to compute the communication strength. At the aggregated level, communication probabilities of all signaling pathways for each pair of cell groups were summarized.

To identify signaling roles (senders or receivers) of cell groups, CellChat adopted the centrality metrics from graph theory and calculated out-degree and in-degree centrality of an intercellular communication network. Regarding the intercellular communication network as a weighted-directed network with the weights as the computed communication probabilities, the out-degree centrality was computed as the sum of communication probabilities of the outgoing signaling from a cell group (treating cells as senders) while the in-degree centrality was computed as the sum of the communication probabilities of the incoming signaling to a cell group (treating cells as receivers).

To identify major signaling changes in mutant testes, a comparison of cell-cell communication network between mutant and wild-type testes was performed. We first compared interaction strength among different cell populations between mutant and wild-type testes. The above analysis summarized the information from the outgoing and incoming signaling together. Next, we compared outgoing and incoming interaction strength of each cell group between mutant and wild-type testes at both the aggregated level and signaling pathway level, which facilitated identifying the cell groups with significant changes in sending or receiving signals and associated signaling pathways that exhibit different signaling patterns.

**CUT&Tag analysis**

For each CUT &Tag dataset, fastp [20] version 0.21.0 was used to profile quality and remove adapters and low-quality reads. Trimmed reads were aligned to *D. melanogaster* reference genome dm6 by Bowtie2[21] version 2.4.3 with the following parameters: --end-to-end --very-sensitive --no-mixed --no-discordant --phred33 -I 10 -X 700. Duplicated reads were removed by Picard (<https://broadinstitute.github.io/picard/>) version 2.21.1 and duplication rate was calculated. Uniquely mapped reads were kept by sambamba [22] version 0.8.0 and reads that aligned to blacklist regions were further filtered. These kept reads were shifted by alignmentSieve from deeptools [23] version 3.1.3 as commonly done for ATAC-seq to account for Tn5 insertion offsets. Specifically, plus-stranded reads were shifted by +4 bp and minus-stranded reads were shifted by -5 bp. Peaks were called by MACS2[24] version 2.2.7.1 with the following parameters: -f BAMPE -g dm --keep-dup all. Replicate reproducibility was assessed by Irreproducibility Discovery Rate (IDR) framework[25] version 2.0.3 and reproducible peaks (IDR < 0.05) were annotated by R package ChIPseeker [26]. Motif enrichment analysis of merged reproducible peaks was performed by findMotifsGenome.pl script of homer (http://homer.ucsd.edu/homer/motif/). HINT-ATAC [27] was used to detect footprinting of de novo and known motifs found by homer in merged reproducible peak regions. To obtain differentially Chinmo binding sites (DBSs) between mutant and wild-type testes, we used R package DiffBind [28] version 2.14.0 to identify statistically significant differentially binding sites (Fold change ≥ 1.5, FDR < 0.05) among all peak regions (sites detected at least in one of the samples) by edgeR statistical framework. Binding signals of differentially binding sites were visualized by R package EnrichedHeatmap [29]. The peak annotation was conducted using the annotatePeak() function of the ChIPseeker R package, which defines the promoter region from -3kb to +3kb of the transcription start site (TSS). Thus, peaks within this distance from the TSS of transcripts are annotated as within the promoter region.

To identify the regulatory nature of Chinmo, Binding and expression target analysis (BETA)[30] is performed. Firstly, each gene is assigned with a regulatory potential score based on the distance between down-regulated Chinmo binding sites in mutant testes and transcription start site. The regulatory potential is calculated as
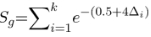
. All binding sites (k) near the transcription start site of the gene (g) within 1000 bp are considered. Δ is the exact distance between a binding site and the TSS proportional to 100 kb (Δ = 0.1 means the exact distance = 10 kb). Next, genes were grouped into three categories based on mutant vs wild-type CySCs differentially expression analysis result, including up-regulated DEGs, down-regulated DEGs and nonsignificant genes in mutant testes. A cumulative distribution function of the three gene groups was generated and a one-tailed Kolmogorov-Smirnov test was used to determine whether up-regulated DEGs group and down-regulated DEGs group differ significantly from the nonsignificant genes group.


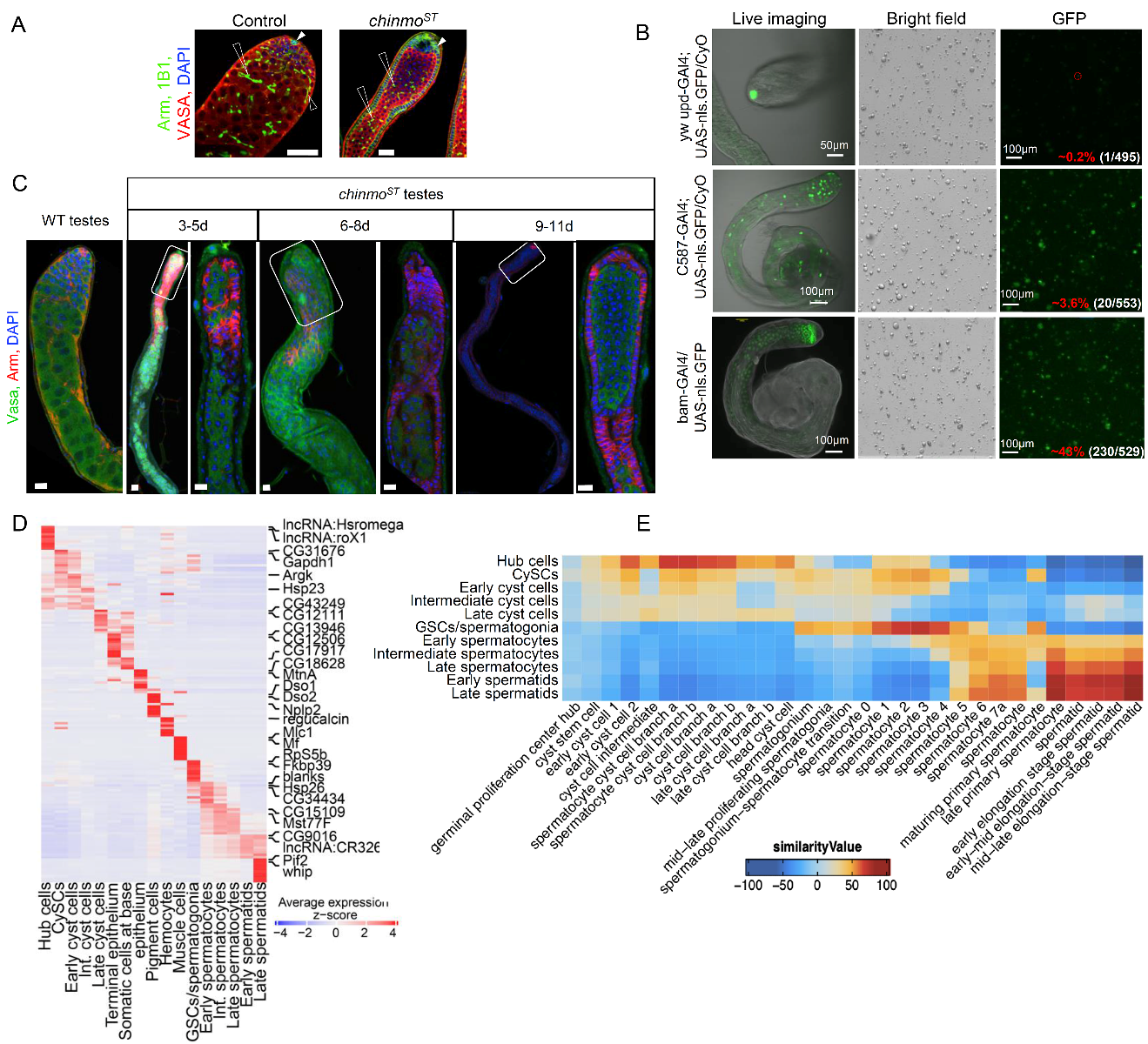


Fig. S1. Single-cell RNA-seq method is robust and reproducible

**A**, One-week-old adult testes immunostained for Vasa (red, germline), Arm (green, somatic cell membrane), 1B1 (green, germ cell fusomes), and DAPI (blue, DNA) in wild-type and *chinmo^ST^* mutant testis. The germ cells in *chinmo^ST^* testes appear to arrest as early male germ cells, resulting in overproliferation of mitotic germ cells (DAPI-bright, with 1B1-positive spherical or short branching fusomes). Scale bars = 20 μm. **B**, Confirmation of single-cell dissociation experiment. Hub cells, cyst cells and germ cells were marked using *upd-Gal4*, *C587-Gal4*, and *bam-Gal4* driving GFP signals, respectively. The percentage refers to target cells recovering from single cell suspensions. **C**, Immunostaining showing *chinmo^ST^* mutant phenotype progression for the whole testis. Scale bars = 20 μm. **D**, Heatmap showing expression (z-score; low: blue, high: red) of the top 10 markers for each of the 17 cell types across the wild-type testes scRNA-seq dataset. Top 2 markers for each cell type are labelled. **E**, Heatmap depicting similarities between cell types identified in our scRNA-seq dataset (rows) and those in the FlyCellAtlas testis snRNA-seq dataset [10] (columns).


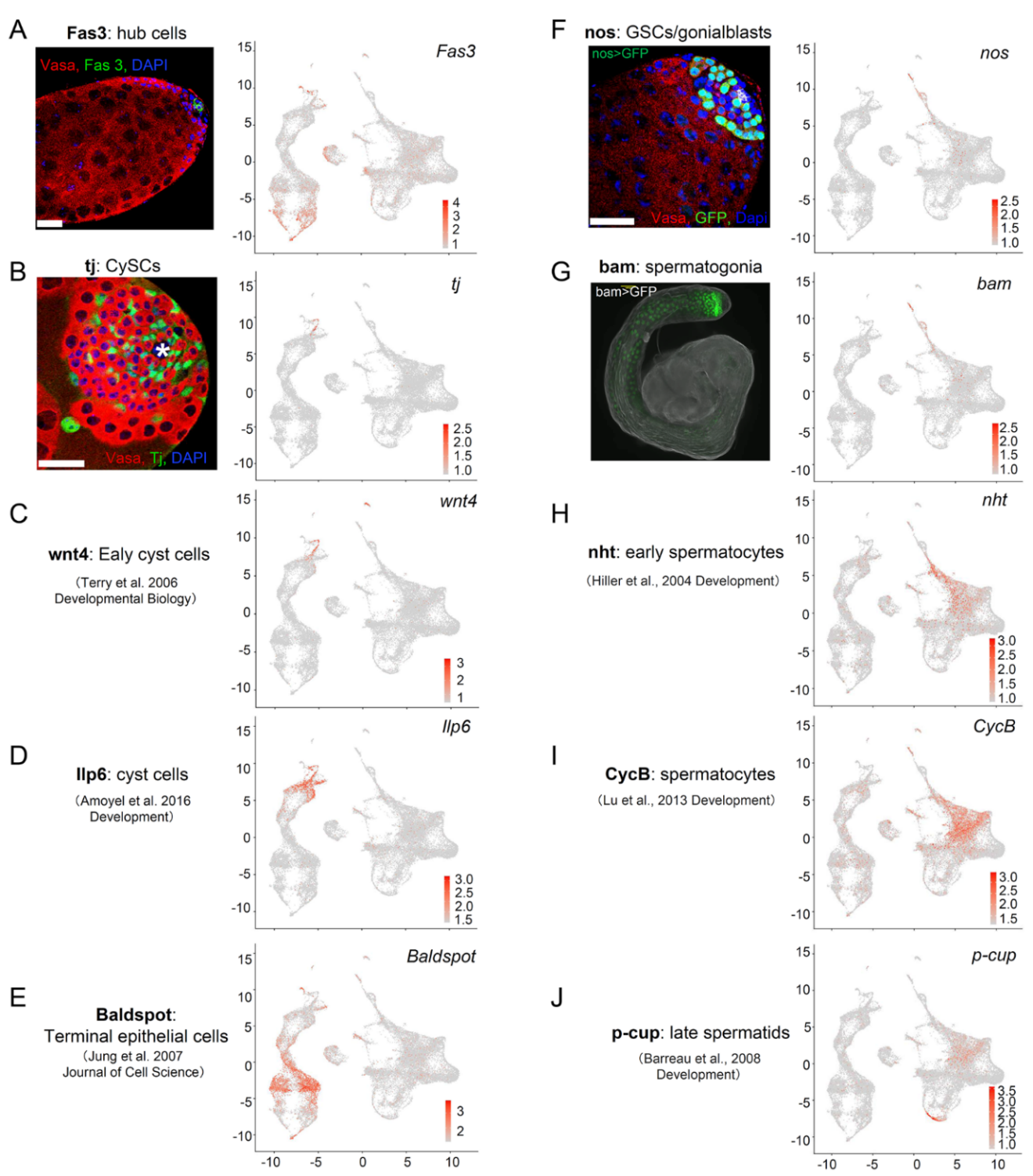


Fig. S2. Single-cell RNA-seq uncovers consistent expression patterns for major testis cell-type marker genes

**A-J**, Immunofluorescence staining and UMAP plots showing consistent expression pattern for somatic cyst cell markers (**A**-**E**): *Fas3* (hub cells and epithelial cells, **A**), *tj* (CySCs, **B**), *Wnt4*[31] (early cyst cells, **C**), *Ilp6*[32] (cyst cells, **D**), *Baldspot*[33] (terminal epithelial cells, **E**). Immunofluorescence staining and UMAP plots showing consistent expression pattern for germ cell markers (**F**-**J**): *nos* (GSCs/gonialblasts, **F**), *bam*-GFP (spermatogonia, **G**), *nht*[34] (early spermatocytes, **H**), *CycB*[35] (spermatocytes, **I**), and *p-cup*[36] (late spermatids, **J**). Red points on the UMAP mark cell populations in which the focal gene is expressed. Scale bars = 20 μm.


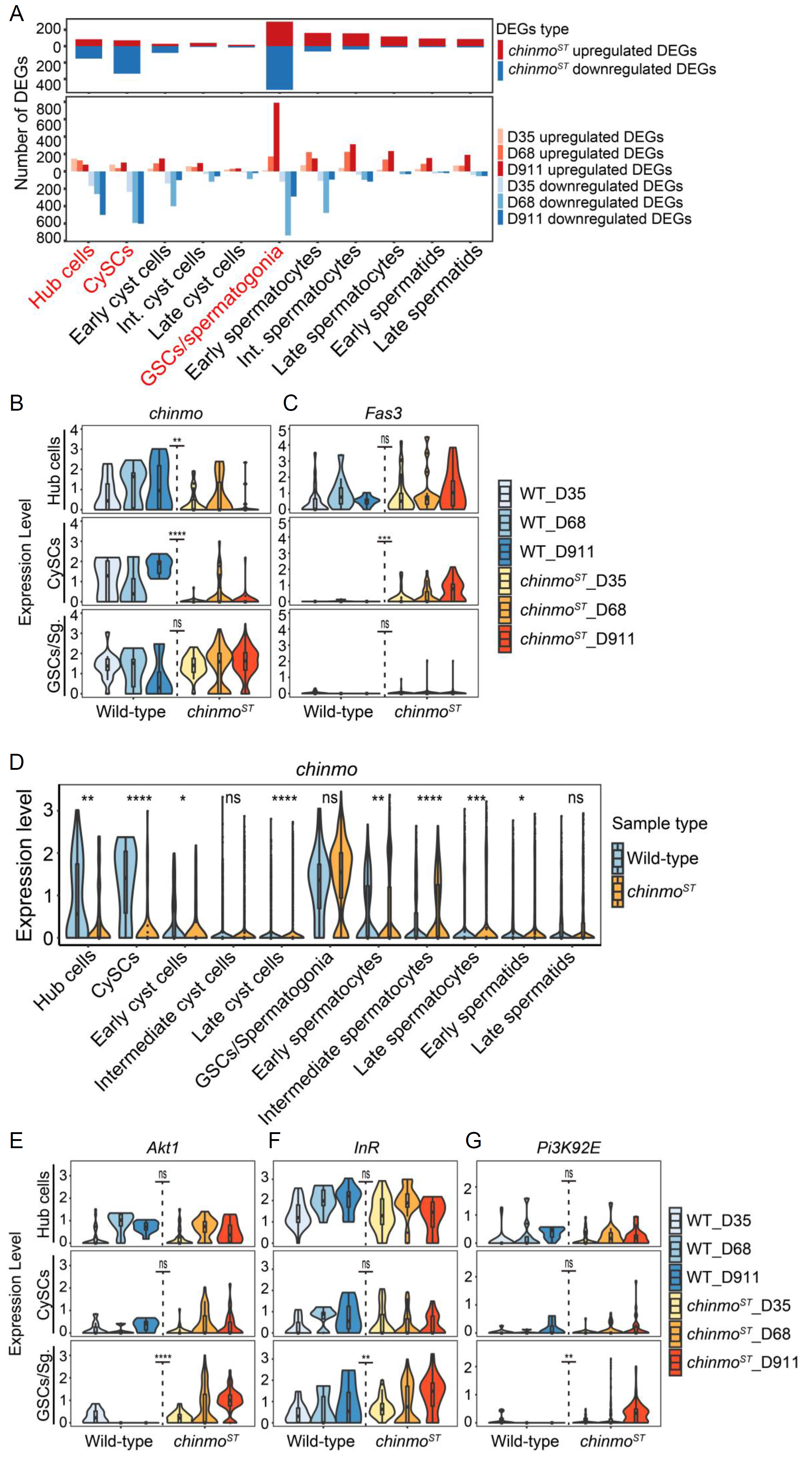


Fig. S3. RNA profile changes in young versus old cells

**A**, Bar graph showing the number of DEGs (Wilcoxon Rank Sum Test p<0.05, absolute value of fold change ≥1.5) in each major *chinmo^ST^* versus wild-type testes cell types over time. Top panel, the number of DEGs obtained from integrating three time points; bottom panel, the number of DEGs at 3–5, 6–8 and 9–11 day-old ***chinmo^ST^*** testes. Red and blue bars represent up- and down-regulated DEGs in *chinmo^ST^* testes, respectively. **B-C,** Violin plots showing the expression changes of *chinmo* (**B**) and *Fas3* (**C**), in hub cells, CySCs, and GSCs/spermatogonia over time. **D**, Violin plots showing the expression changes of *chinmo* in each major *chinmo^ST^* versus wild-type testes cell type. **E-G**, Violin plots showing the expression changes of *Akt1* (**E**), *InR* (**F**) and *Pi3K92E* (**G**) in hub cells, CySCs, and GSCs/spermatogonia over time. Sg, Spermatogonia. Int, intermediate. WT, wild-type. D35, 3-5 day-old. D68, 6-8 day-old. D911, 9-11 day-old. Differential expression analysis was performed by Wilcoxon rank sum test (**, p < 0.01; ***, p < 0.001; ****, p < 0.0001; ns, nonsignificant).


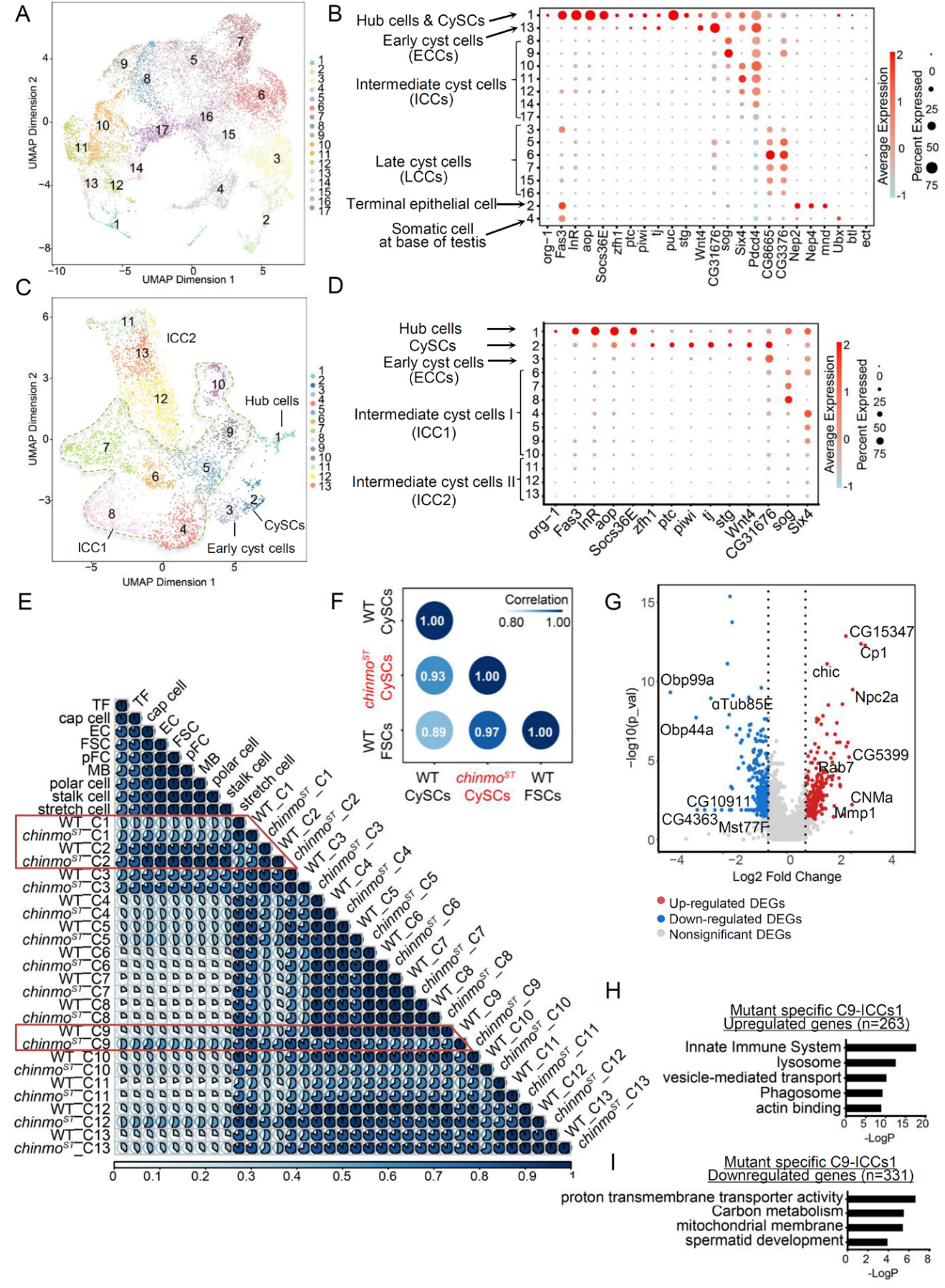


Fig. S4. *chinmo^ST^*-specific CySCs and follicle-like cells display a molecular basis of feminization on the transcriptome level

**A-B**, UMAP plot (**A**) of somatic cells subclustering with cluster annotation and dot plot (**B**) showing gene expression profiles of selected somatic cell markers in the integrated scRNA-seq datasets. Dot color represents scaled average gene expression, and dot size represents percentage of expressed cells within a cluster. **C-D**, UMAP plot (**C**) of cyst cells subclustering (excluding late cyst cells) with cluster and cell type annotation (the dotted line marks subclusters of ICCs1), and dot plot (**D**) showing gene expression profiles of selected markers in the integrated scRNA-seq datasets. Dot color represents scaled average gene expression. Dot size represents percentage of expressed cells within a cluster. **E**, Pairwise Pearson correlation of pseudobulk transcriptomes among all the 13 wild-type and *chinmo^ST^* testes cyst cell clusters and wild-type ovarian somatic cells [37]. Color intensity and filled pie chart area are proportional to the Pearson correlation coefficients. Outlines were added to emphasize the remarkable increases of correlations with ovarian somatic cells for *chinmo^ST^* hub cells, CySCs and cluster-9 follicle-like cells, compared to wild-type testes. **F**, Pie charts showing Pearson correlation coefficients of pseudobulk transcriptomes among *chinmo^ST^* CySCs, wild-type CySCs and wild-type ovarian FSCs [37]. Color intensity is proportional to the Pearson correlation coefficients. **G**, Volcano plot of DEGs between *chinmo^ST^* and wild-type C9-ICCs1. The x-axis represents the log^2^ fold change and the y-axis represents -log^10^ (p-value). Genes with Wilcoxon rank sum test p-value < 0.05 and absolute value of fold change ≥ 1.5 are considered DEGs, while others are considered as nonsignificant DEGs. **H**-**I**, GO terms enriched for up- (**H**) and down-regulated DEGs (**I**) in *chinmo^ST^* mutant-specific C9-ICCs1, respectively.


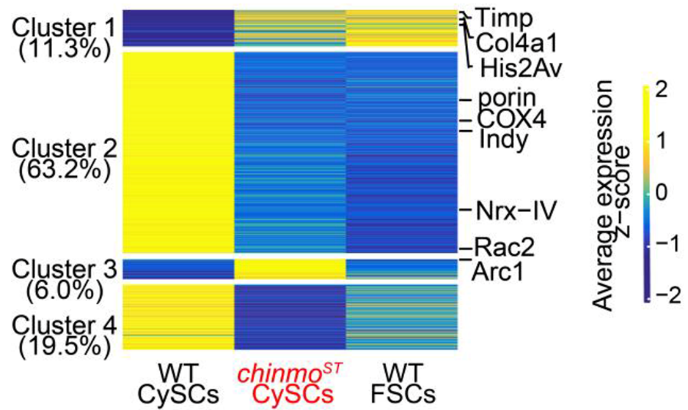


Fig. S5. Down-regulation of male-biased genes and up-regulation of female-biased genes in *chinmo^ST^* CySCs suggest feminization of *chinmo*-depleted CySCs

Heatmap showing expression of all 416 DEGs (*chinmo^ST^* CySCs versus wild-type CySCs, Wilcoxon Rank Sum Test p<0.05, absolute fold change ≥1.5) in wild-type CySCs, *chinmo^ST^* CySCs and wild-type ovarian FSCs [37] , the female counterpart of CySCs. Color intensity indicates gene expression, calculated as the z-transformed average expression (z-score range is -2 to 2). Female-biased genes are defined as genes displaying ≥ 1.5-fold expression in wild-type ovary germ cells compared to wild-type testis germ cells; male-biased genes are defined as genes displaying ≥ 1.5-fold expression in wild-type testis germ cells compared to wild-type ovary germ cells. Wilcoxon Rank Sum Test p-value < 0.05. Cluster 1 contains female-biased genes that are upregulated in *chinmo^ST^* CySCs; cluster 2 contains male-biased genes that are downregulated in *chinmo^ST^* CySCs. Clusters 3 and 4 contain mutant-specific DEGs that may not relate to sex conversion features of *chinmo^ST^* CySCs.


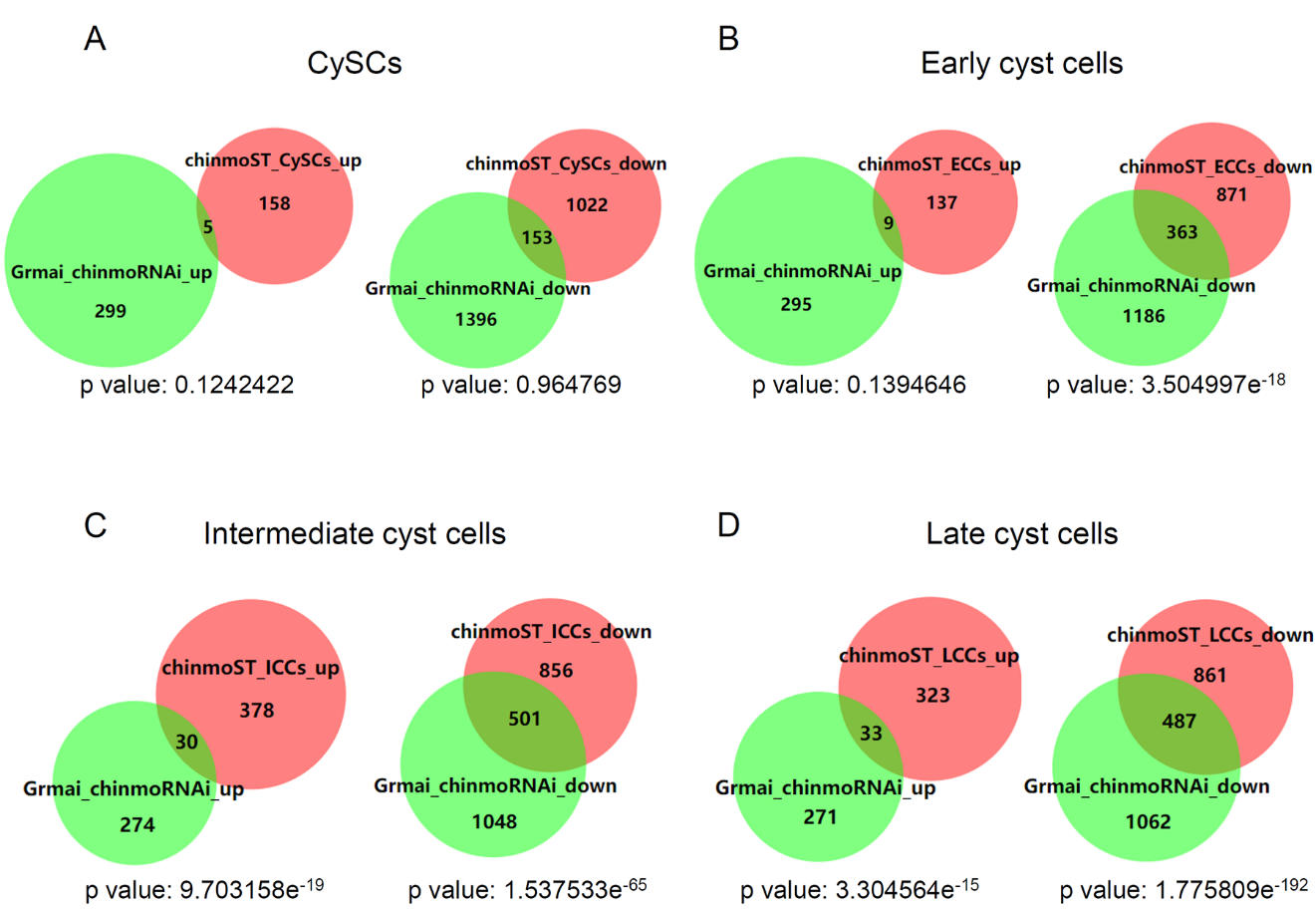


Fig. S6. Significant overlaps of DEGs between Grmai et al. 2021 and *chinmo^ST^* cyst cell populations

**A-D**, Pie charts indicating the count of overlapping DEGs in *chinmo* mutant versus wild-type somatic cells between this study and a recent investigation employing bulk RNA-seq on sorted *chinmo*-depleted somatic cells[38]. Green segments represent the numbers of up- and down-regulated DEGs in Grmai et al. 2021[38]. Red segments indicate the numbers of up- and down-regulated DEGs between each annotated *chinmo^ST^* cyst cell population and wild-type cyst cell populations in our study (Wilcoxon Rank Sum Test p < 0.05, absolute fold change ≥ 1.2). The designated cyst cell populations include CySCs, cyst stem cells (**A**); ECCs, early cyst cells (**B**); ICCs, intermediate cyst cells (**C**); LCCs, late cyst cells (**D**). Up, upregulated genes. Down, downregulated genes. Differential analysis was performed by hypergeometric test. The reportedly upregulated genes such as *beta-Spec*, *DE-cad* (*shg*), *mirr*, and *pyd* in bulk somatic cells[38] are particularly upregulated within intermediate cyst cells.


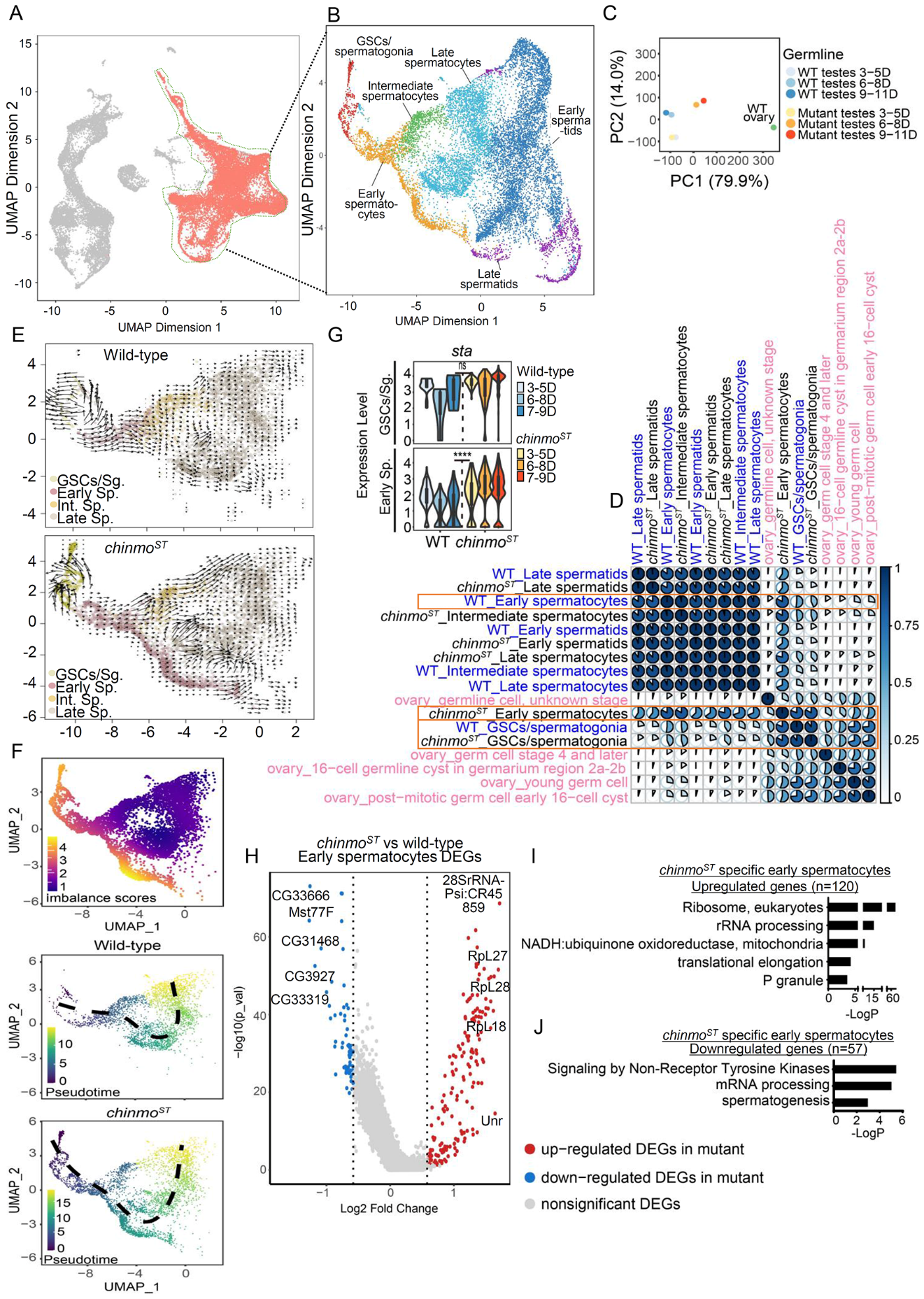


Fig. S7. Disruption of gene expression in GSCs/spermatogonia contributes to the feminization of early spermatocytes in the *chinmo^ST^* testis

**A**, UMAP plot highlighting germline cells (red points) in the integrated scRNA-seq dataset, which were subset for further clustering. **B**, UMAP plot showing germline cells subclustering with annotated cell types. **C,** Two-dimensional principal component analysis (PCA) plot of pseudobulk transcriptomes of germline cells in wild-type testes (blue), *chinmo^ST^* testes (orange) and wild-type ovaries[10] (green). Color shading indicates days of adulthood. The first principal component (PC1) axis explains the largest variation (79.9%) of bulk transcriptomes across all samples so that the transcriptome distances among samples can be inferred based on the locations along the PC1 axis. The transcriptome of *chinmo^ST^* mutant germline cells deviates from wild-type testes and more closely resembles wild-type ovaries with age. **D**, Pie charts showing pairwise Pearson correlation of pseudobulk transcriptomes among all the germline cell types from wild-type testes, *chinmo^ST^* testes and wild-type ovaries [10]. Color intensity and filled pie chart area are proportional to the Pearson correlation coefficients. Outlines were added to emphasize the changes of correlations with ovarian germ cells for *chinmo^ST^* GSCs/spermatogonia and early spermatocytes, compared to wild-type testes. **E**, RNA velocities that are projected into UMAP embedding of wild-type and *chinmo^ST^* testis germ cells, respectively. The arrows’ directions indicate extrapolated future states and the lengths of arrows indicate the amount of transcriptome changes. Long arrows imply large changes in gene expression and rapid differentiation while short arrows are associated with terminally differentiated cells maintaining homeostasis. **F**, Top: UMAP plot showing imbalance scores of germline cells in the integrated scRNA-seq dataset. The imbalance score measures the imbalance between the local and global distributions of condition labels. GSCs/spermatogonia and early spermatocytes show high imbalance scores because they are *chinmo^ST^* testes-specific GSCs/spermatogonia and early spermatocytes population. Bottom: Trajectories from GSCs/spermatogonia to late spermatocytes in wild-type testes and *chinmo^ST^* testes. Color indicates inferred pseudo-time by slingshot. Unsupervised RNA velocity analysis was performed and the differentiation trajectories of wild-type and *chinmo^ST^* germ cells was compared. A part of early spermatocyte cells remains “stuck” between intermediate and late spermatocytes along the mutant trajectory. These findings are consistent with our anatomical observation that a subset of *chinmo^ST^* testes germ cells is arrested in the early stage of spermatogenesis [2], which suggests that GSCs/spermatogonia in *chinmo^ST^* testes proliferate substantially and their descendant spermatogonia-to-spermatocyte-transition cells are indeed arrested at an early stage and unable to differentiate into mature sperm cells. **G**, Violin plots showing expression of *sta* in germ cells over time. Differential expression analysis is performed by Wilcoxon rank sum test (*, p < 0.05; **, p < 0.01; ****, p < 0.0001; ns, nonsignificant). **H**, Volcano plot of DEGs between *chinmo^ST^* and wild-type early spermatocytes. The x-axis represents the log^2^ fold change and the y-axis represents -log^10^ (p-value). Genes with Wilcoxon rank sum test p-value < 0.05 and absolute value of fold change ≥ 1.5 are considered DEGs, while others are considered nonsignificant DEGs. Top 5 up- and down-regulated DEGs are labelled. **I-J**, GO terms enriched for up- (**I**) and down-regulated DEGs (**J**) in *chinmo^ST^* early spermatocytes, respectively. WT, wild-type. Sg, spermatogonia. Sp, spermatocytes.


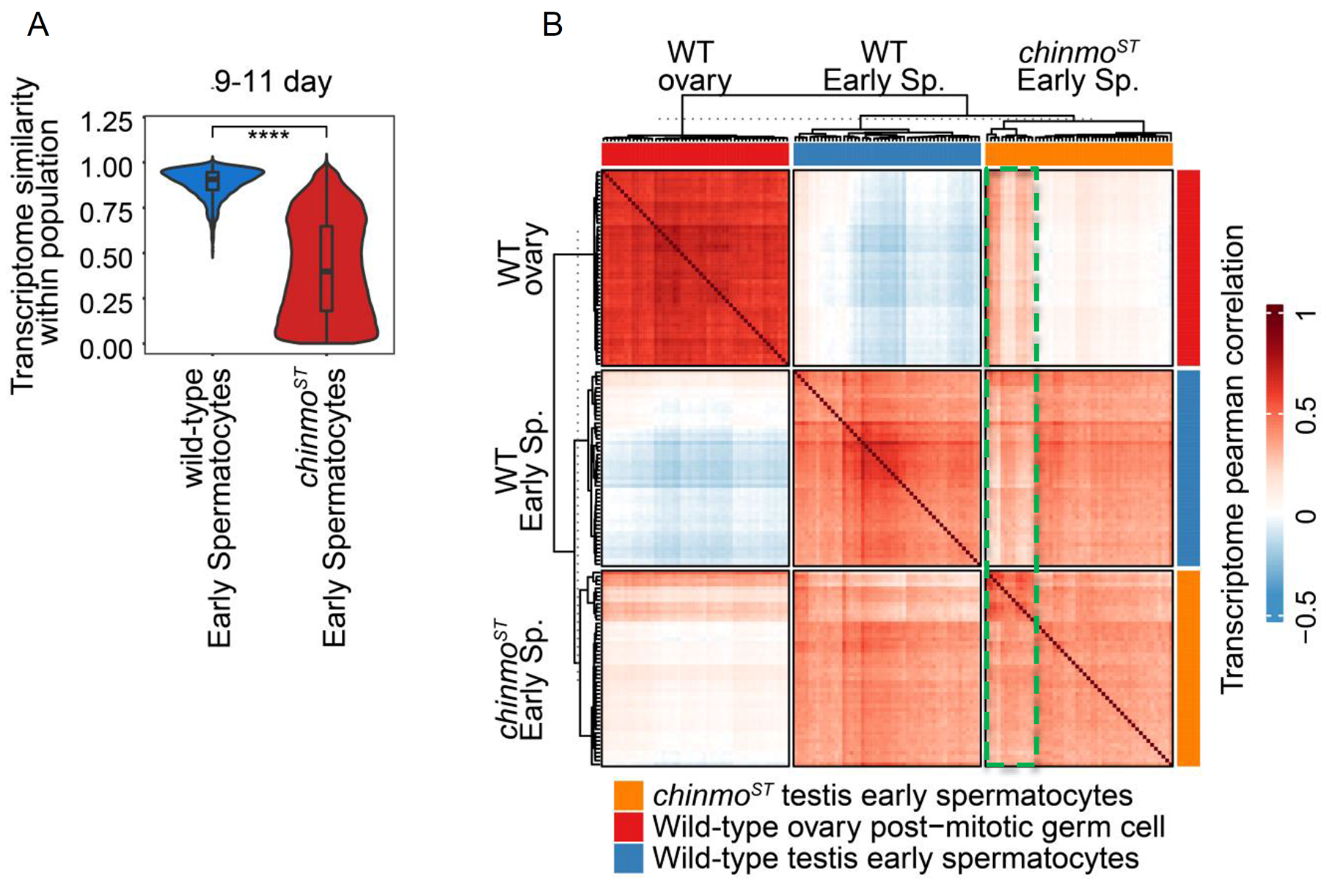


Fig. S8. Transcriptomic heterogeneity in *chinmo^ST^* early spermatocytes reflects partial sex-transformation within the cell population

**A**, Violin plots displaying comparisons of whole transcriptome similarities within 9-11 day-old early spermatocyte populations of wild-type testes and *chinmo^ST^* testes. **B**, Heatmap showing the transcriptome similarities of sex biased genes among individual cells of wild-type and *chinmo^ST^* early spermatocytes, in addition to ovarian post-mitotic germ cells [10]. Color intensity represents the Pearson correlation coefficients. A dashed outline highlights the subpopulation of cells exhibiting increased similarity with ovarian germ cells and decreased male identity.


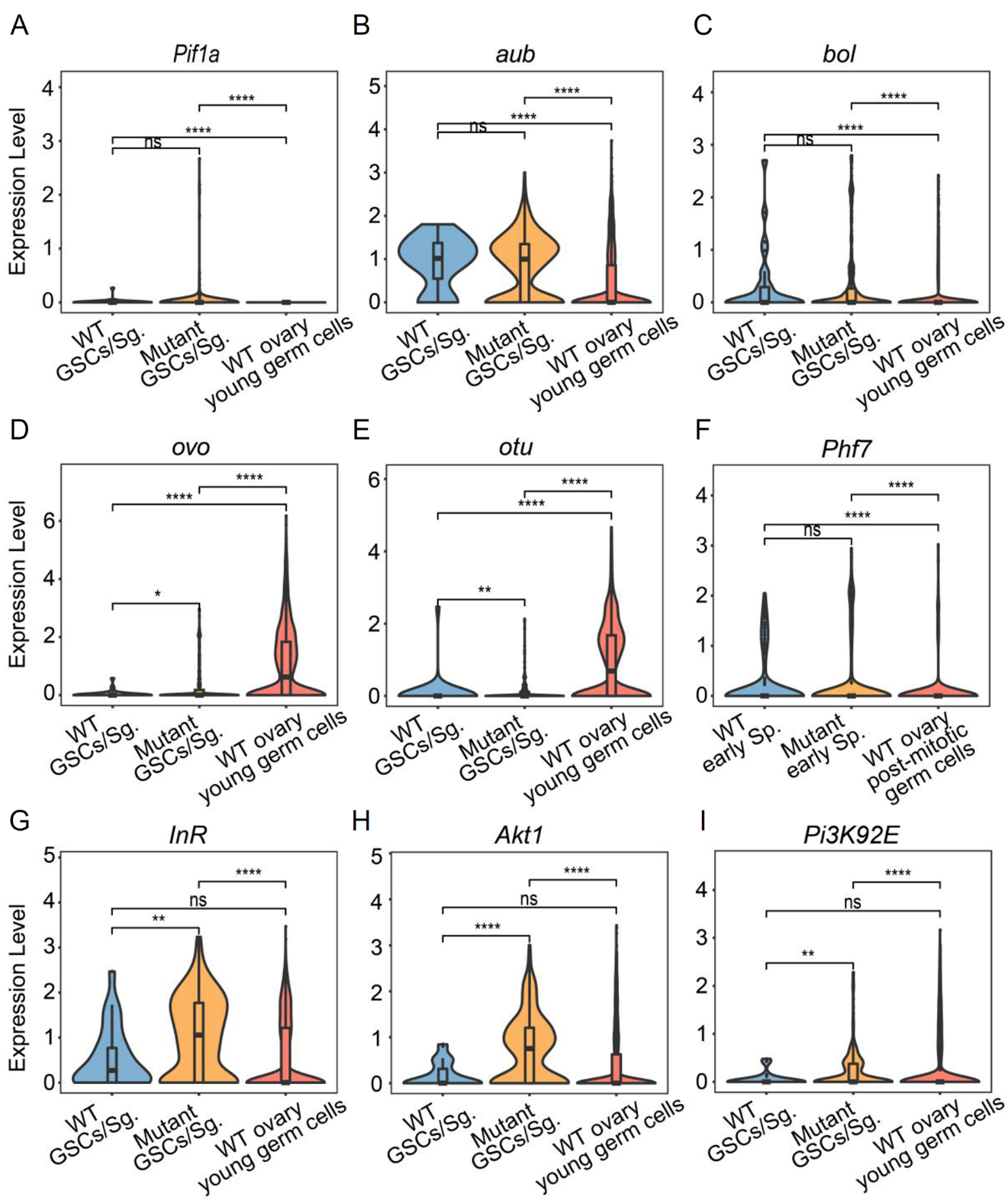


Fig. S9. Comparative RNA expression analysis of specific genes in germ cells across wild-type testes, *chinmo^ST^* testes, and wild-type ovaries

**A-C**, Violin plots showing expression profiles of genes retaining male expression pattern, including *Pif1a*, *aub*, and *bol* in GSCs/Spermatogonia of *chinmo^ST^* testes, compared to that of wild-type testes and young germ cells of wild-type ovaries. **D-E**, Violin plots showing expression profiles of genes involved in oocytes differentiation such as *ovo* and *otu*, being intermediate in GSCs/Spermatogonia of *chinmo^ST^* testes, compared to that of wild-type testes and young germ cells of wild-type ovaries. **F**, Violin plot showing expression of gene *Phf7* retaining male expression pattern in early spermatocytes of *chinmo^ST^* testes, compared to that of wild-type testes and post-mitotic germ cells of wild-type ovaries. **G-I**, Violin plots demonstrating the gene expression profiles of components associated with the Insulin receptor signaling pathway, namely *InR* (**G**), *Akt1* (**H**), and *Pi3k92E* (**I**) in GSCs/Spermatogonia of wild-type testes, *chinmo^ST^* testes, and young germ cells of wild-type ovaries. Differential expression analysis is performed by Wilcoxon rank sum test (***, p < 0.001; *****, p < 0.00001; ns, nonsignificant). WT, wild-type. Sg, spermatogonia. Sp, spermatocytes.


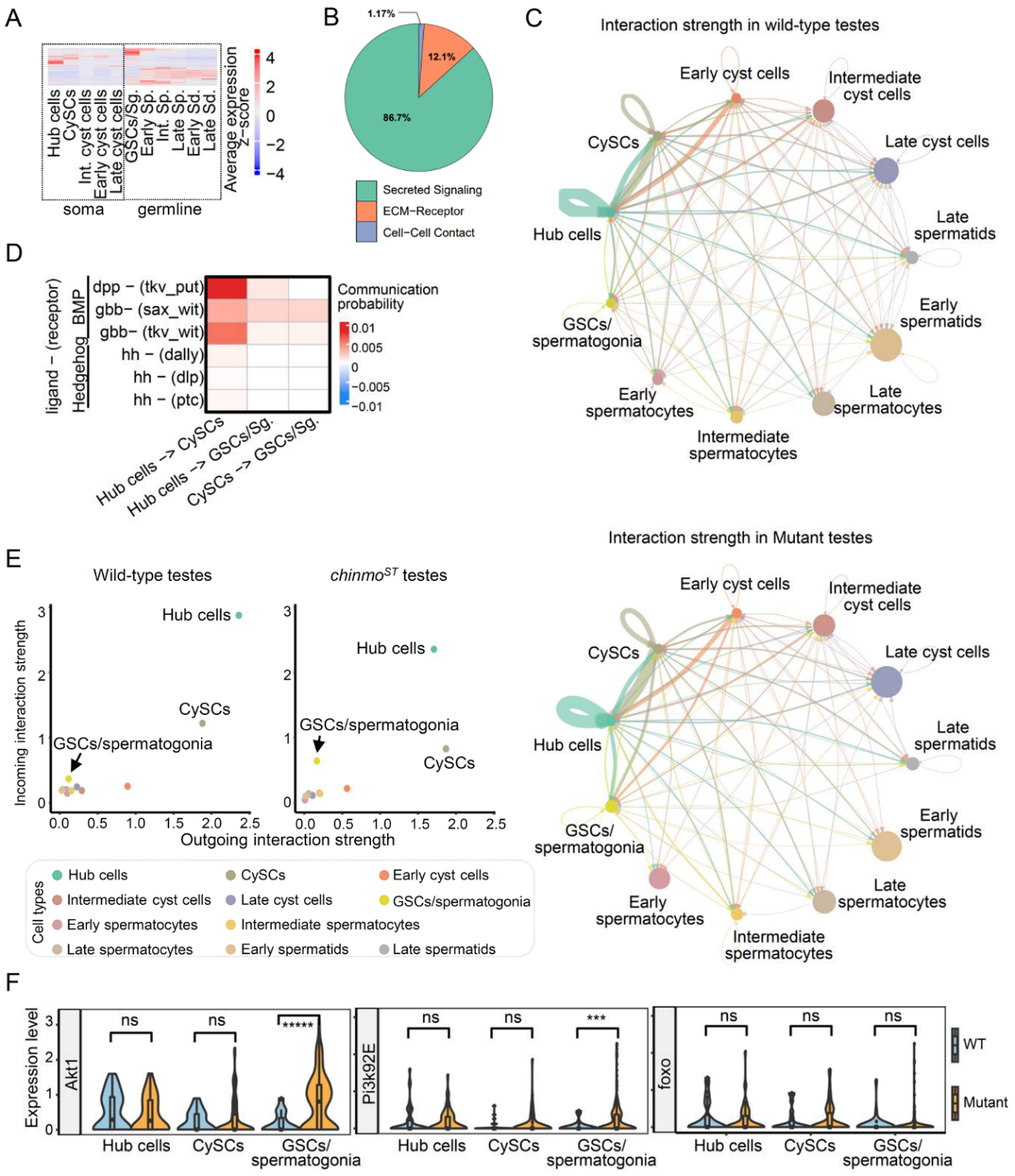


Fig. S10. Active cell-cell interactions are predicted in testis stem cell niche and Insulin signaling mediates soma-germline crosstalk

**A**. Heatmap showing expression (z-score; low: blue, high: red) of the 410 *Drosophila* genes associated with male sterility in the Flybase [39, 40], within each of the 11 major soma and germ cell types in the wild-type testes scRNA-seq dataset. **B**, Pie chart showing proportion of signaling molecule interaction types curated for *Drosophila* in this study. 256 molecular interactions were included: 86.7% of secreted signaling interactions, 12.1% of extracellular matrix (ECM)-receptor interactions and 1.17% of cell-cell contact interactions. **C**, Circle plots showing inferred cell-cell communication networks for wild-type and *chinmo^ST^* testes. Intercellular signaling strength between any two cell types is shown. Circle color indicates cell types and circle sizes proportional to the number of cells in each cell group. Widths of edge lines are proportional to the interaction strength. **D**, Heatmap showing communication probability of each identified ligand-receptor in BMP and Hedgehog signaling pathway for wild-type testes. The BMP ligands Gbb and Dpp are sent from hub cells and CySCs to GSCs/spermatogonia, and the Hh ligands are sent from hub cells to CySCs as expected. Darker shade indicates higher communication probability. **E**, Two-dimensional plot showing incoming and outgoing interaction strengths for each major cell type in wild-type and *chinmo^ST^* testes, respectively. Dot color represents cell types. **F**, Violin plot showing gene expression of Insulin receptor signaling components, including *Akt1*, *Pi3k92E* and *foxo* in niche cell populations (hub cells, GSC/spermatogonia and CySCs) of wild-type and *chinmo^ST^* testes. Differential expression analysis is performed by Wilcoxon rank sum test (***, p < 0.001; *****, p < 0.00001; ns, nonsignificant). Sg, Spermatogonia; Sp, Spermatocytes. Sd. Spermatids.


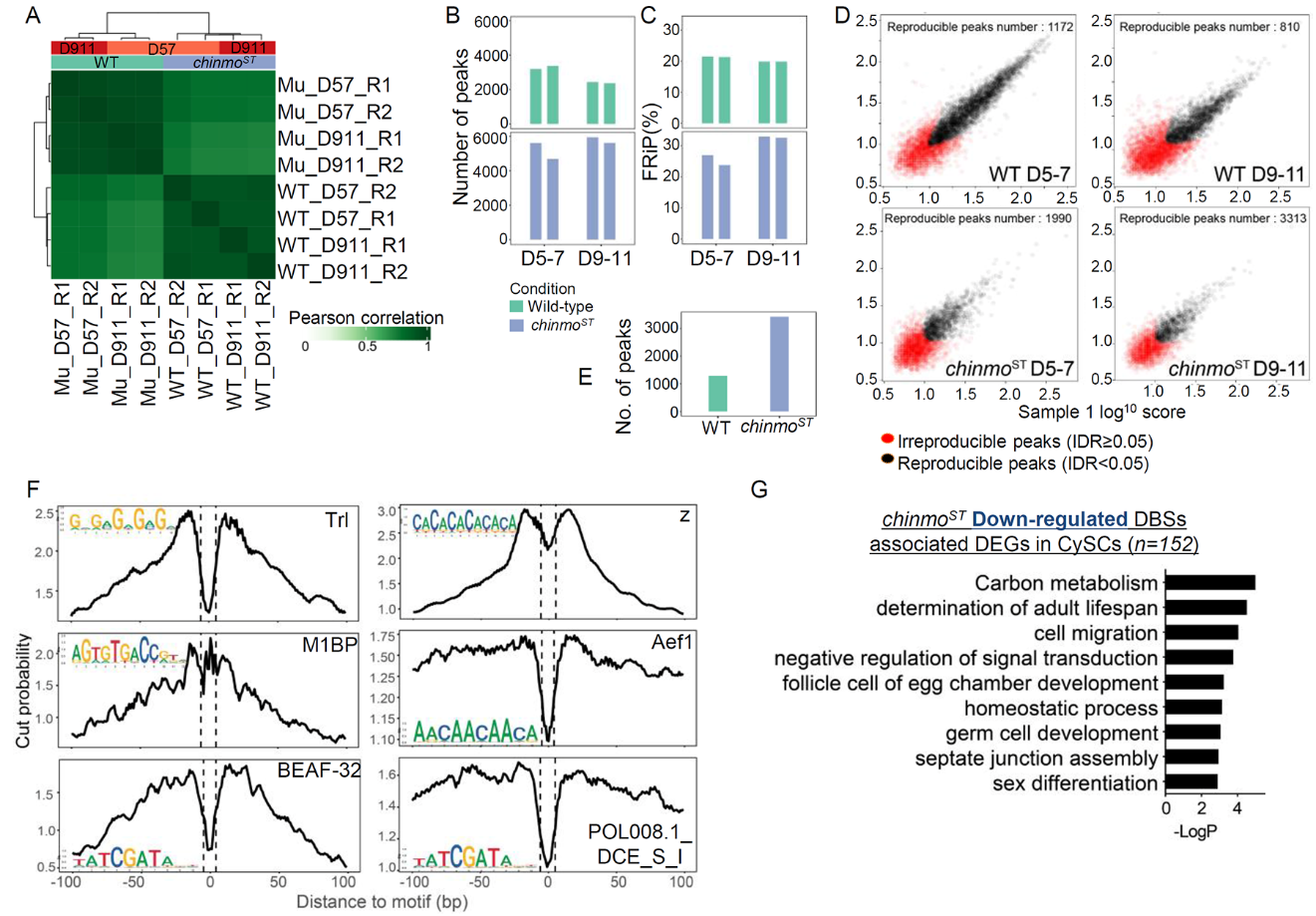


Fig. S11. Chinmo CUT&Tag sequencing is robust and reproducible

**A**, Heatmap showing correlations of read counts in peaks among all CUT&Tag samples. Replicates cluster together appropriately. Darker shade indicates stronger correlation. R, replicate. **B**. Bar plot showing the number of called peaks for each replicate of 5–7 and 9–11 day-old wild-type and *chinmo^ST^* testes. Color indicates the genotype of testis. **C**, Bar plot showing fraction of reads in peaks for each replicate of 5–7 and 9–11 day-old wild-type and *chinmo^ST^* testes. Color indicates the genotype of testis. **D**, Scatter plots of signal scores of peaks that overlap in the pair of replicates for 5–7 and 9–11 day-old wild-type and *chinmo^ST^* testes. Black points represent pairs of peaks passing an IDR threshold of 0.05 (reproducible peaks); red points represent pairs of peaks that do not pass the IDR threshold of 0.05 (irreproducible peaks). **E**, Bar plot showing the number of merged reproducible Chinmo binding peaks (by merging reproducible peaks of different time points for the same condition) in wild-type and *chinmo^ST^* testes, respectively. **F,** Aggregate footprints for Trl, z, M1BP, Aef1, BEAF-32 and POL008.1_DCE_S_I (motifs shown) generated over binding sites within wild-type reproducible Chinmo binding peaks. **G**, Top GO terms enriched for down-regulated Chinmo DBSs-associated DEGs in *chinmo^ST^* CySCs. WT, wild-type. D35, 3-5 day-old. D68, 6-8 day-old. D911, 9-11 day-old.


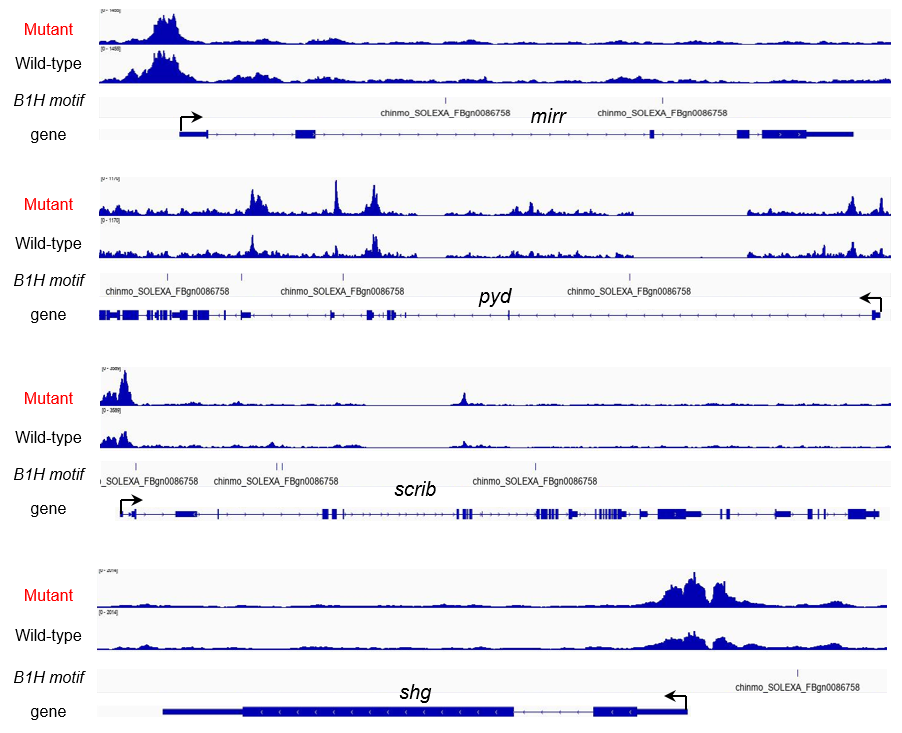


Fig. S12. Chinmo binding sites on previously documented Chinmo targets via Chinmo CUT&Tag and B1H screening methods

Genome tracks depicting the exemplary Chinmo direct targets, such as *mirr*, *pyd*, *scrib*, and *shg*, identified through both B1H and CUT&Tag methods. The binding sites of Chinmo identified based on these two distinct methodologies overlap on these Chinmo targets.


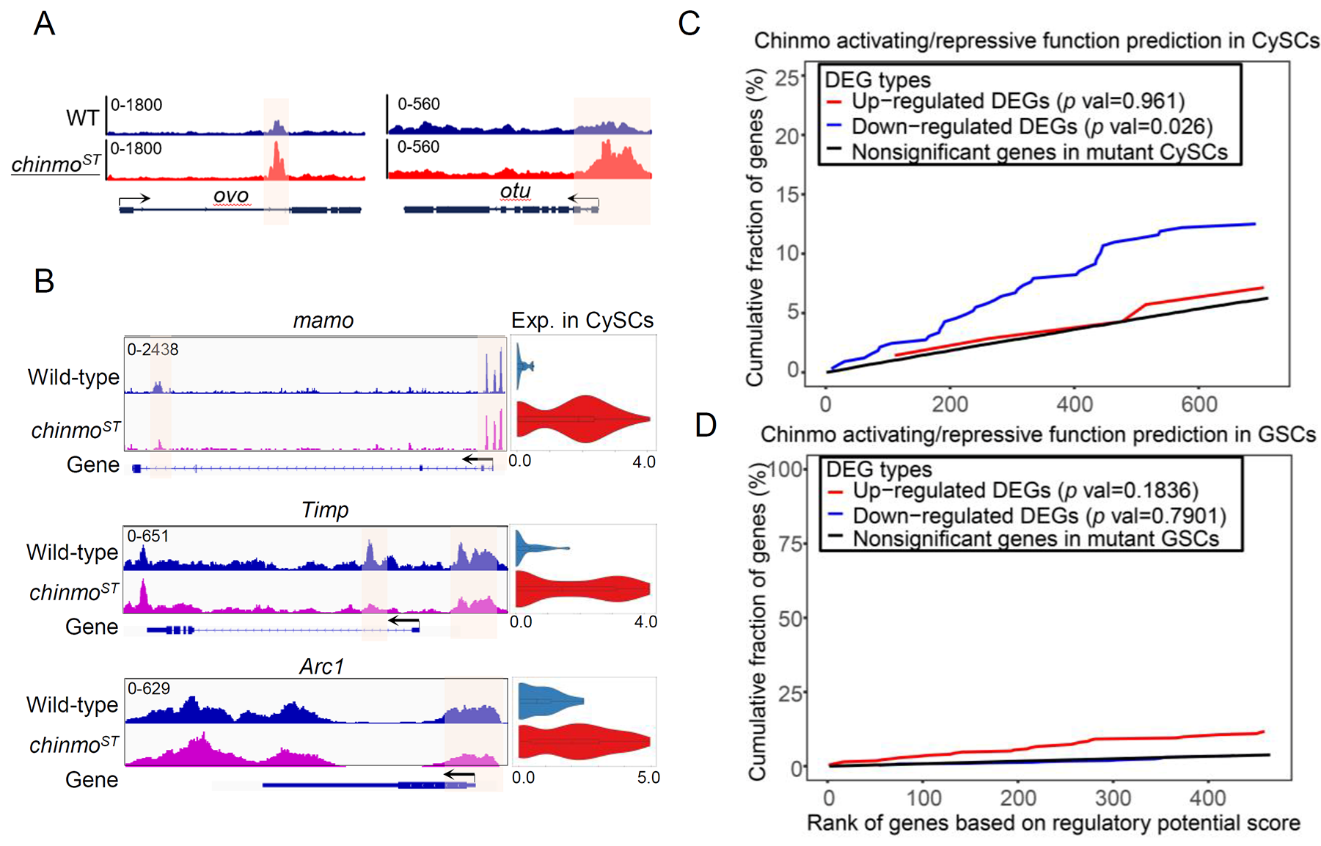


Fig. S13. Chinmo acts as both a transcriptional repressor and a transcriptional activator

**A**. Genome tracks from CUT&Tag analysis reveal enhanced Chinmo binding on the promoter region of female germline determination factors, *ovo* and *otu*, in *chinmo^ST^* testes compared to wild-type testes. **B**, Genome tracks for exemplary Chinmo direct targets identified by CUT&Tag, including the receptor tyrosine kinase regulator *mamo*, the ECM remodeling factor *Timp* and the vesicle transporter *Arc1*. Violin plots on the right showing expression of these genes in wild-type and *chinmo^ST^* testes, respectively. **C-D**, Plot showing cumulative distribution for up-, down-regulated DEGs and nonsignificant genes in *chinmo^ST^* mutant-specific CySCs (**C**) and GSCs/Spermatogonia (**D**) after ranking genes by regulatory potential score. One-tailed Kolmogorov-Smirnov test is used to determine whether the up- or down-regulated DEGs group differ significantly from the nonsignificant gene group. p-value is shown in the upper left. Using the Binding and Expression Target Analysis (BETA) framework [30], we investigated Chinmo's role as an activator or repressor. In CySCs with reduced Chinmo expression, downregulated genes exhibit significantly higher regulatory potential scores compared to upregulated and nonsignificant genes (**C**). Conversely, in GSCs/spermatogonia with slightly increased Chinmo expression, upregulated genes demonstrate higher regulatory potential scores compared to downregulated and nonsignificant genes (**D**). Despite previous indications of Chinmo acting primarily as a transcriptional repressor, our findings suggest that Chinmo predominantly functions as a transcriptional activator in both somatic and germ cells.

Supplementary Table S1.

ScRNA-seq cell type annotation and testis single-cell datasets comparisons. An .xlsx workbook file consists of gene markers used for cell type annotation of single-cell data, comparisons across recently published testis single-cell datasets, the cell number of somatic cell types, and the cell number of germline cell types, respectively.

Supplementary Table S2.

Manually curated signaling molecule interaction database for *Drosophila*. An .xlsx workbook file containing four worksheets that list detailed information of ligand-receptor interaction, receptor complex, cofactors and all genes in *Drosophila*. All the four worksheets serve as input for updating CellChatDB. See sheet titled “README” for more information and definition of column headings.

Supplementary Table S3.

Inferred cellular communication networks at the level of ligand-receptor pair for control and mutant testes. An .xlsx workbook file containing two worksheets that list detailed information of inferred ligand-receptor pair interaction between two cell types in control and mutant testes, respectively. See sheet titled “README” for more information and definition of column headings.

**Supplementary** **Table S4. (Related to Figure 5) RNAi knock down of Insulin pathway components in GSCs induces the follicle cell-like phenotype and germ cell overproliferation.** Exp, experiment; CT, control.

| Genotype | Group | Days of RNAi induction ^*^ | % testes with follicle-like cell phenotype and overproliferated germ cells | % testes with overproliferated germ cells |
| --- | --- | --- | --- | --- |
| *UAS-InR^R418P^/+; nos-gal4/+* | Exp | 10-13 ^*^ | 52 (24/46) |  |
|  |  | 16-19 ^*^ | 62 (26/42) |  |
|  |  | 23-26 ^*^ | 65 (26/40) |  |
| *UAS-InR^A1325D^/+; nos-gal4/+* | Exp | 16-19 ^*^ | 29 (14/49) | 14 (7/49) |
|  |  | 23-26 ^*^ | 42 (28/66) | 15 (10/66) |
| *nos-gal4/UAS-Akt1* | Exp | 10-13 ^*^ | 48 (31/64) |  |
| *y w;;nos-gal4*  *UAS-GFP/+; nos-gal4/+*  *UAS-InR^R418P^*  *UAS-InR^A1325D^*  *UAS-Akt1* | CT | 10-13 ^*^ | 0 (n>10) | 0 (n>10) |
|  |  | 16-19 ^*^ | 0 (n>10) | 0 (n>10) |
|  |  | 23-26 ^*^ | 0 (n>10) | 0 (n>10) |
| ^*^ Flies were raised at 25 ˚C. After eclosion, adult flies were shifted to 31 ˚C for 1-3 weeks to induce RNAi. | | | | |

Supplementary Table S5. (Related to Figure 5) RNAi knock down of Insulin pathway components in GSCs induces the follicle cell-like phenotype and germ cell overproliferation. Exp, experiment; CT, control.

| Genotype | Group | Days of RNAi induction ^*^ | % testes with follicle-like cell phenotype and overproliferated germ cells | % testes with overproliferated germ cells |
| --- | --- | --- | --- | --- |
| *UAS-InR^R418P^/+; nos-gal4/+* | Exp | 10-13 ^*^ | 50 (10/20) |  |
|  |  | 23-26 ^*^ | 64 (7/11) |  |
| *UAS-InR^A1325D^/+; nos-gal4/+* | Exp | 16-19 ^*^ | 25 (2/8) | 13 (1/8) |
|  |  | 23-26 ^*^ | 42 (5/12) | 25 (3/12) |
| *nos-gal4/UAS-Akt1* | Exp | 10-13 ^*^ | 44 (8/18) |  |
| *y w;;nos-gal4*  *UAS-GFP/+; nos-gal4/+*  *UAS-InR^R418P^*  *UAS-InR^A1325D^*  *UAS-Akt1* | CT | 10-13 ^*^ | 0 (n>10) | 0 (n>10) |
|  |  | 16-19 ^*^ | 0 (n>10) | 0 (n>10) |
|  |  | 23-26 ^*^ | 0 (n>10) | 0 (n>10) |
| ^*^ Flies were raised at 25 ˚C. After eclosion, adult flies were shifted to 29 ˚C for 1-3 weeks to induce RNAi. | | | | |

**Supplementary** **Table S6.** **(Related to Figure 5) Knock-down of InR in the GSC lineage partially rescues the *chinmo^ST^* phenotype.** Exp, experiment; CT, control; N, the total number of testes sampled; d, days.

| Use *nos-Gal4* to Express | | Group |  | % Testes with FasIII^+^ Somatic Aggregates | | | | | |
| --- | --- | --- | --- | --- | --- | --- | --- | --- | --- |
|  |  |  | Notes | 0 d | 3 d | 5 d | 7 d | 9 d | 11 d |
| *chinmo^ST^/CyO; nos-Gal4 or UAS-InR RNAi /TM3* | | CT1 | Mild-I  Mild-II  Severe  N= | 0  16 | 0  31 | 0  52 | 0  28 | 0  20 | 0  27 |
| *chinmo^ST^/CyO; nos-Gal4/UAS-InR RNAi* | | CT2 | Mild-I  Mild-II  Severe  N= | 0  35 | 0  30 | 0  55 | 0  21 | 0  23 | 0  21 |
| *chinmo^ST^; nos-Gal4/UAS-InR RNAi* | | Exp | Mild-I  Mild-II  Severe  N= | 3  0  0  35 | 25  0  0  89 | 31  32  19  108 | 15  5  49  65 | 17  12  60  84 | 19  13  64  216 |
| *chinmo^ST^; nos-Gal4 or UAS-InR RNAi /TM3* | | CT3 | Mild-I  Mild-II  Severe  N= | 16  0  0  37 | 38  0  0  68 | 26  22  34  107 | 3  17  72  86 | 6  18  65  62 | 1  12  87  91 |
|  | All flies were raised at 25 ˚C. After eclosion, adult flies were shifted to 31 ˚C for 1-2 weeks to induce RNAi. *UAS-InR* *RNAi* = *UAS-InR^HMS03166^* *RNAi*.  Mild-I phenotype [2]: Fas3^+^ aggregates around hub cells.  Mild-II phenotype [2]: Fas3^+^ aggregates at the distal 1/5-2/3 of *chinmo^ST^* testes.  Severe phenotype [2]: Fas3^+^ aggregates at the distal 2/3 or whole *chinmo^ST^* testes. | | | | | | | | |

Supplementary Table S7. (Related to Figure 5) Knock-down of InR in the GSC lineage partially rescues the *chinmo^ST^* phenotype. Exp, experiment; CT, control; N, the total number of testes sampled; d, days.

| Use *nos-Gal4* to Express | | Group |  | % Testes with FasIII^+^ Somatic Aggregates | | | | | |
| --- | --- | --- | --- | --- | --- | --- | --- | --- | --- |
|  |  |  | Notes | 0 d | 3 d | 5 d | 7 d | 9 d | 11 d |
| *chinmo^ST^/CyO; nos-Gal4 or UAS-InR RNAi /TM3* | | CT1 | Mild-I  Mild-II  Severe  N= | 0  17 | 0  16 | 0  20 | 0  16 | 0  20 | 0  20 |
| *chinmo^ST^/CyO; nos-Gal4/UAS-InR RNAi* | | CT2 | Mild-I  Mild-II  Severe  N= | 0  16 | 0  15 | 0  23 | 0  20 | 0  23 | 0  19 |
| *chinmo^ST^; nos-Gal4/UAS-InR RNAi* | | Exp | Mild-I  Mild-II  Severe  N= | 3  0  0  29 | 21  0  0  19 | 29  25  25  28 | 17  6  56  18 | 16  16  60  25 | 15  10  70  20 |
| *chinmo^ST^; nos-Gal4 or UAS-InR RNAi /TM3* | | CT3 | Mild-I  Mild-II  Severe  N= | 4  0  0  26 | 22  0  0  18 | 31  19  31  26 | 11  16  63  19 | 3  16  74  31 | 4  14  82  22 |
|  | All flies were raised at 25 ˚C. After eclosion, adult flies were shifted to 29 ˚C for 1-2 weeks to induce RNAi. *UAS-InR* *RNAi* = *UAS-InR^HMS03166^* *RNAi*.  Mild-I phenotype [2]: Fas3^+^ aggregates around hub cells.  Mild-II phenotype [2]: Fas3^+^ aggregates at the distal 1/5-2/3 of *chinmo^ST^* testes.  Severe phenotype [2]: Fas3^+^ aggregates at the distal 2/3 or whole *chinmo^ST^* testes. | | | | | | | | |

Supplementary Table S8 (separate file).

Chinmo CUT&Tag binding sites and the expression profiles of Chinmo binding sites-associated targets. An .xlsx workbook file containing three worksheets that list detailed information of merged wild-type testis reproducible peaks, merged mutant reproducible peaks and differential peaks between wild-type testes and *chinmo^ST^* testes, a list of 152 direct targets of Chinmo within CySCs, respectively. See sheet titled “README” for more information and definition of column headings.

**SI References**

1. Kai T, Spradling A. An empty Drosophila stem cell niche reactivates the proliferation of ectopic cells. *Proc Natl Acad Sci U S A*. 2003; **100**(8): 4633-4638. doi: 10.1073/pnas.0830856100

2. Ma Q, Wawersik M, Matunis EL. The Jak-STAT target Chinmo prevents sex transformation of adult stem cells in the Drosophila testis niche. *Dev Cell*. 2014; **31**(4): 474-486. doi: 10.1016/j.devcel.2014.10.004

3. Matunis E, Tran J, Gönczy P *et al.* punt and schnurri regulate a somatically derived signal that restricts proliferation of committed progenitors in the germline. *Development*. 1997; **124**(21): 4383-4391. doi: 10.1242/dev.124.21.4383

4. Wu YC, Chen CH, Mercer A *et al.* Let-7-complex microRNAs regulate the temporal identity of Drosophila mushroom body neurons via chinmo. *Dev Cell*. 2012; **23**(1): 202-209. doi: 10.1016/j.devcel.2012.05.013

5. Tang Y, Geng Q, Chen D *et al.* Germline Proliferation Is Regulated by Somatic Endocytic Genes via JNK and BMP Signaling in Drosophila. *Genetics*. 2017; **206**(1): 189-197. doi: 10.1534/genetics.116.196535

6. Kaya-Okur HS, Wu SJ, Codomo CA *et al.* CUT&Tag for efficient epigenomic profiling of small samples and single cells. *Nat Commun*. 2019; **10**(1): 1930. doi: 10.1038/s41467-019-09982-5

7. Zheng GX, Terry JM, Belgrader P *et al.* Massively parallel digital transcriptional profiling of single cells. *Nat Commun*. 2017; **8**: 14049. doi: 10.1038/ncomms14049

8. Stuart T, Butler A, Hoffman P *et al.* Comprehensive Integration of Single-Cell Data. *Cell*. 2019; **177**(7): 1888-1902.e1821. doi: 10.1016/j.cell.2019.05.031

9. Raz AA, Vida GS, Stern SR *et al.* Emergent dynamics of adult stem cell lineages from single nucleus and single cell RNA-Seq of Drosophila testes. *eLife*. 2023; **12**: e82201. doi: 10.7554/eLife.82201

10. Li H, Janssens J, De Waegeneer M *et al.* Fly Cell Atlas: A single-nucleus transcriptomic atlas of the adult fruit fly. *Science*. 2022; **375**(6584): eabk2432. doi: 10.1126/science.abk2432

11. Witt E, Benjamin S, Svetec N *et al.* Testis single-cell RNA-seq reveals the dynamics of de novo gene transcription and germline mutational bias in Drosophila. *Elife*. 2019; **8**. doi: 10.7554/eLife.47138

12. Mahadevaraju S, Fear JM, Akeju M *et al.* Dynamic sex chromosome expression in Drosophila male germ cells. *Nature Communications*. 2021; **12**(1): 892. doi: 10.1038/s41467-021-20897-y

13. Yu G, Wang LG, Han Y *et al.* clusterProfiler: an R package for comparing biological themes among gene clusters. *Omics*. 2012; **16**(5): 284-287. doi: 10.1089/omi.2011.0118

14. Tenenbaum D. KEGGREST: Client-side REST access to KEGG. *R package version 1261*. 2019.

15. La Manno G, Soldatov R, Zeisel A *et al.* RNA velocity of single cells. *Nature*. 2018; **560**(7719): 494-498. doi: 10.1038/s41586-018-0414-6

16. de Bézieux HR, Van den Berge K, Street K *et al.* Trajectory inference across multiple conditions with condiments: differential topology, progression, differentiation, and expression. *bioRxiv*. 2021: 2021.2003.2009.433671. doi: 10.1101/2021.03.09.433671

17. Liu Y, Li JSS, Rodiger J *et al.* FlyPhoneDB: an integrated web-based resource for cell-cell communication prediction in Drosophila. *Genetics*. 2022; **220**(3). doi: 10.1093/genetics/iyab235

18. Jin S, Guerrero-Juarez CF, Zhang L *et al.* Inference and analysis of cell-cell communication using CellChat. *Nat Commun*. 2021; **12**(1): 1088. doi: 10.1038/s41467-021-21246-9

19. Jin S, Guerrero-Juarez CF, Zhang L *et al.* Inference and analysis of cell-cell communication using CellChat. *Nature Communications*. 2021; **12**(1): 1088. doi: 10.1038/s41467-021-21246-9

20. Chen S, Zhou Y, Chen Y *et al.* fastp: an ultra-fast all-in-one FASTQ preprocessor. *Bioinformatics*. 2018; **34**(17): i884-i890. doi: 10.1093/bioinformatics/bty560

21. Langmead B, Salzberg SL. Fast gapped-read alignment with Bowtie 2. *Nat Methods*. 2012; **9**(4): 357-359. doi: 10.1038/nmeth.1923

22. Tarasov A, Vilella AJ, Cuppen E *et al.* Sambamba: fast processing of NGS alignment formats. *Bioinformatics*. 2015; **31**(12): 2032-2034. doi: 10.1093/bioinformatics/btv098

23. Ramírez F, Ryan DP, Grüning B *et al.* deepTools2: a next generation web server for deep-sequencing data analysis. *Nucleic Acids Res*. 2016; **44**(W1): W160-165. doi: 10.1093/nar/gkw257

24. Zhang Y, Liu T, Meyer CA *et al.* Model-based analysis of ChIP-Seq (MACS). *Genome Biol*. 2008; **9**(9): R137. doi: 10.1186/gb-2008-9-9-r137

25. Li Q, Brown JB, Huang H *et al.* Measuring reproducibility of high-throughput experiments. *The Annals of Applied Statistics*. 2011; **5**(3): 1752-1779, 1728.

26. Yu G, Wang LG, He QY. ChIPseeker: an R/Bioconductor package for ChIP peak annotation, comparison and visualization. *Bioinformatics*. 2015; **31**(14): 2382-2383. doi: 10.1093/bioinformatics/btv145

27. Li Z, Schulz MH, Look T *et al.* Identification of transcription factor binding sites using ATAC-seq. *Genome Biol*. 2019; **20**(1): 45. doi: 10.1186/s13059-019-1642-2

28. Rory Stark GB. DiffBind: differential binding analysis of ChIP-Seq peak data. <http://bioconductor.org/packages/release/bioc/vignettes/DiffBind/inst/doc/DiffBind.pdf>. 2011.

29. Gu Z, Eils R, Schlesner M *et al.* EnrichedHeatmap: an R/Bioconductor package for comprehensive visualization of genomic signal associations. *BMC Genomics*. 2018; **19**(1): 234. doi: 10.1186/s12864-018-4625-x

30. Wang S, Sun H, Ma J *et al.* Target analysis by integration of transcriptome and ChIP-seq data with BETA. *Nat Protoc*. 2013; **8**(12): 2502-2515. doi: 10.1038/nprot.2013.150

31. Terry NA, Tulina N, Matunis E *et al.* Novel regulators revealed by profiling Drosophila testis stem cells within their niche. *Dev Biol*. 2006; **294**(1): 246-257. doi: 10.1016/j.ydbio.2006.02.048

32. Amoyel M, Hillion K-H, Margolis SR *et al.* Somatic stem cell differentiation is regulated by PI3K/Tor signaling in response to local cues. *Development (Cambridge, England)*. 2016; **143**(21): 3914-3925. doi: 10.1242/dev.139782

33. Jung A, Hollmann M, Schäfer MA. The fatty acid elongase NOA is necessary for viability and has a somatic role in Drosophila sperm development. *J Cell Sci*. 2007; **120**(Pt 16): 2924-2934. doi: 10.1242/jcs.006551

34. Hiller M, Chen X, Pringle MJ *et al.* Testis-specific TAF homologs collaborate to control a tissue-specific transcription program. *Development*. 2004; **131**(21): 5297-5308. doi: 10.1242/dev.01314

35. Lu C, Kim J, Fuller MT. The polyubiquitin gene Ubi-p63E is essential for male meiotic cell cycle progression and germ cell differentiation in Drosophila. *Development*. 2013; **140**(17): 3522-3531. doi: 10.1242/dev.098947

36. Barreau C, Benson E, Gudmannsdottir E *et al.* Post-meiotic transcription in Drosophila testes. *Development*. 2008; **135**(11): 1897-1902. doi: 10.1242/dev.021949

37. Rust K, Byrnes LE, Yu KS *et al.* A single-cell atlas and lineage analysis of the adult Drosophila ovary. *Nat Commun*. 2020; **11**(1): 5628. doi: 10.1038/s41467-020-19361-0

38. Grmai L, Harsh S, Lu S *et al.* Transcriptomic analysis of feminizing somatic stem cells in the Drosophila testis reveals putative downstream effectors of the transcription factor Chinmo. *G3 (Bethesda)*. 2021; **11**(4). doi: 10.1093/g3journal/jkab067

39. Gramates LS, Agapite J, Attrill H *et al.* FlyBase: a guided tour of highlighted features. *Genetics*. 2022; **220**(4). doi: 10.1093/genetics/iyac035

40. Ibaraki K, Nakatsuka M, Ohsako T *et al.* A cross-species approach for the identification of Drosophila male sterility genes. *G3 (Bethesda)*. 2021; **11**(8). doi: 10.1093/g3journal/jkab183
